# Profiling peripheral immune cells in Parkinson's disease: A scoping review

**DOI:** 10.1177/1877718X261479505

**Published:** 2026-10-08

**Authors:** Sherilyn Junelle Recinto, Janna E Jernigan Posey, Nadejda Lefter, Zagorka Vitic, Morten Øgelund Overgaard, Lin Liu, Andrew JM Howden, Klaus Eyer, Marina Romero-Ramos, Malú Gámez Tansey, Jo Anne Stratton

**Affiliations:** 1 Department of Neurology and Neurosurgery, 55981 Montreal Neurological Institute-Hospital, McGill University, Montreal, Canada; 2 Aligning Science across Parkinson's (ASAP) Collaborative Research Network, Chevy Chase, MD, USA; 3 Department of Neurology, 12250 Indiana University School of Medicine, Stark Neuroscience Research Institute, Indianapolis, IN, USA; 4 Division of Cell Signaling and Immunology, School of Life Sciences, 3042 University of Dundee, Dundee, UK; 5 Department of Biomedicine, 1006 Aarhus University, Aarhus, Denmark; 6 Lundbeck Foundation Parkinson's disease Research Center (PACE), 1006 Aarhus University Hospital, Aarhus, Denmark; 7 Department of Microbiology and Immunology, 5620 McGill University, Montreal, Canada

**Keywords:** Parkinson's disease, PBMCs, immune system, innate, adaptive

## Abstract

Parkinson's disease (PD) is increasingly recognized as a multi-system disorder with immune dysregulation extending beyond the central nervous system. Although numerous studies have examined peripheral immune alterations in people with PD, findings remain heterogeneous and difficult to reconcile. To clarify the current landscape, we conducted a comprehensive scoping review of human studies profiling peripheral blood immune cells in PD. Following PRISMA-ScR guidelines, we systematically screened the literature and curated studies reporting *in vivo* and *ex vivo* immune characterizations from PD patients. Eligible studies based on pre-defined criteria were assessed for patient demographics and clinical variables, experimental and analytical approaches, and reported immune outcomes. Our synthesis reveals a steady expansion and diversification of peripheral immune cell research in PD especially over the last decade. Deep immunophenotyping identifies convergent signatures across *in vivo* studies of both innate and adaptive compartments, including expanded pro-inflammatory T-cell subsets, altered monocyte subset distributions, increased cytotoxic natural killer cells and neutrophil-to-lymphocyte ratio, and dysregulated pathways related to immune activation, chemotaxis, mitochondrial function, and autophagy-lysosomal processes. Stimulation-based *ex vivo* assays further demonstrate recurrent T-cell hyper-responsiveness in PD, whereas myeloid cell responses are more variable and context dependent. Critically, this review highlights substantial variability and under-reporting in study design, which impeded our ability to make strong conclusions relating to many aspects of PD peripheral immunity.

## Plain language summary

Changes in Blood Immune Cells in Parkinson’s Disease: What Research Has Found So Far

Parkinson's disease (PD) is best known for causing movement problems such as tremors and stiffness. However, growing evidence shows that it also affects the body's immune system, not only in the brain but throughout the body. Many researchers have studied immune cells in the blood of people with PD, but the results have not always been consistent or easy to compare. To better understand what is known, we reviewed published studies that examined immune cells in the blood of people living with PD. We focused on research that directly measured immune cells in patients, either by analyzing them as they are in the body or by testing how they respond to stimulation in the laboratory. We found that research in this area has increased significantly over the past decade. Across many studies, several common patterns emerged. People with PD often show signs of a more “activated” immune system. Certain types of T cells (a key component of the adaptive immunity) appear to be more inflammatory. Changes were also seen in innate immune populations, including monocytes, neutrophils and natural killer cells, which are involved in detecting and responding to threats. Some studies also reported disruptions in basic cell functions, such as energy production, waste removal, and movement of immune cells within the body. When immune cells were stimulated in laboratory experiments, T cells from people with PD frequently showed stronger responses than those from healthy individuals, whereas results from monocytes were more variable. Overall, our review highlights common immune changes in PD but also shows that differences in study design and reporting make it difficult to draw firm conclusions. More standardized research is needed to better understand how the immune system contributes to PD.

## Introduction

As the second most common and fastest growing neurodegenerative disorder, Parkinson's Disease (PD) represents a major public health concern.^1,2^ It is projected to impact 25.2 million people worldwide by 2050, representing a 112% increase from 2021.^3^ People with PD (PwPD) experience a wide range of symptoms including sensory defects, affective disorders, cognitive decline or dementia, sleep disturbances, autonomic dysfunction that can impact the cardiovascular, gastrointestinal, genitourinary, and thermoregulatory systems, and characteristic motor impairments such as tremor, bradykinesia, rigidity, and postural instability that worsen with disease duration.^4^ Treatment options so far are limited to symptomatic alleviation and do not halt or reverse the progression of the disease.

Historically, PD research has focused on neuropathological changes within the central nervous system (CNS); however, PD is now recognized as a multi-system disorder involving both central and peripheral immune alterations. A growing body of work implicates peripheral inflammation, gut-brain axis perturbations, and immune cell infiltration in disease initiation and progression, with Braak staging supporting a model in which pathology may spread from the gastrointestinal tract to the brain. Immune dysregulation has been documented across clinical, imaging, and molecular studies, including microglial dysfunction,^5,6^ elevated cytokines and chemokines,^7,8^ blood-brain-barrier disruption,^9,10^ and inflammatory changes in blood and cerebrospinal fluid (CSF).^11–13^ Moreover, circulating immune cell analyses indicate that inflammatory activity may peak early in disease to decrease or fail at later stages.^14–21^ Collectively, these findings suggest that immune alterations are not merely secondary to neurodegeneration but may play causal roles in PD pathogenesis.

Despite this convergence, the PD immune literature remains fragmented and, at times, inconsistent. While changes in the amounts of cytokines/chemokines in biofluids may reflect the participation of peripheral immunity in disease mechanisms, their interpretation is complicated by pre-analytical and biological variability, including assay sensitivity and environmental factors, such as infections, circadian rhythms and diet.^22–25^ Furthermore, these measurements alone provide limited insight into the cellular sources and underlying molecular pathways driving immune alterations in PD. To overcome these challenges, it is crucial to directly assess changes in immune cell populations – their frequencies, phenotypes, and activation states. Such studies offer greater resolution for elucidating specific immune cell subsets involved, and the use of functional stimulation-based *ex vivo* assays may uncover immune phenotypes that could otherwise be challenging to decipher at baseline and could be affected by exogenous factors.

That said, as with the study of any complex immunological disease, immune cell profiles and inferred mechanisms vary across studies, likely due to both biological and technical causes. Disease heterogeneity, patient demographics, disease stages/subtypes, differences in assay platforms, sample handling, analytical methods, and more, contribute to challenges in drawing definitive conclusions relating alterations in peripheral immunity in PD. Here we aimed to perform a comprehensive scoping review as an essential first step toward identifying common patterns, methodological gaps, and opportunities for standardization within the PD peripheral immune cell literature. We conducted a comprehensive search using the PubMed database by employing keywords and Medical Subject Headings (MeSH) including those related to “Parkinson's disease”, “periphery” and “inflammation”. Where some studies were not identified based on these search criteria, additional studies were included based on expert reviewer knowledge. Titles, abstracts and full texts were screened, and data were extracted by multiple independent researchers using a curated set of criteria such as patient demographics, experimental design and study rigor. We highlight reproducible immune features across studies, while emphasizing the need for standardized reporting practices and cell-based analytical approaches to advance our understanding of immune dysregulation in PD. This work may ultimately help facilitate the development of targeted immunomodulatory therapeutic strategies and biomarker discovery.

## Results

### Systematic survey of literature on peripheral immunity in Parkinson's disease

To ensure a comprehensive and unbiased assessment of existing research on peripheral immune cell alterations in PD, a scoping review was conducted in accordance with the Preferred Reporting Items for Systematic reviews and Meta-Analyses extension for Scoping Reviews (PRISMA-ScR) guidelines (Figure 1A). A predefined search strategy was applied to the PubMed database (Table S1), and all retrieved records underwent title and abstract screening by two reviewers. Eligibility was determined using pre-specified inclusion criteria. As such, studies reporting only inflammatory mediators in biofluids (*e.g.,* plasma/serum, CSF, saliva, tear or exosomes), studies pertaining exclusively to the microbiome, and studies of other synucleinopathies (*e.g.,* dementia with Lewy Bodies, multiple system atrophy) were excluded. At level-1 screening, we also excluded papers describing non-human primary cells or CNS-resident immune cells and glia. Moreover, full-text articles were assessed, and we excluded any reviews, meta-analyses, clinical trials, non-English articles, and records without easily accessible full-texts. Following expert review by five principal investigators, we further excluded studies lacking matched healthy controls, as well as genome-wide association studies (GWAS), and studies focused solely on platelets or red blood cells. Twenty-four additional studies were identified through reference screening and expert recommendation. Ultimately, a total of 292 articles were included in the qualitative synthesis, encompassing patient demographics, methodological characteristics, study design features, and a summary of principal findings.

**Figure 1.**
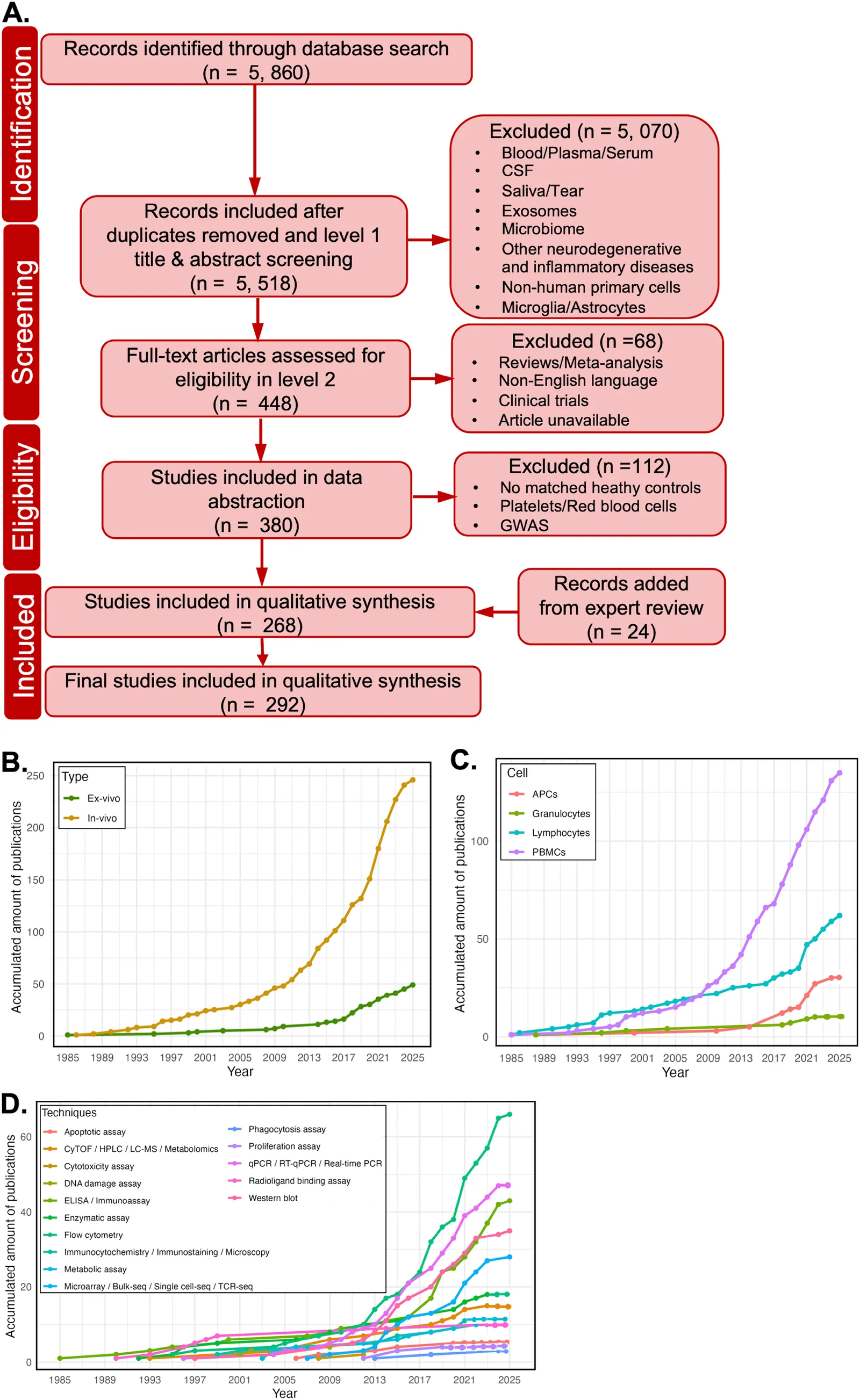
Longitudinal overview of the growth, scope, and methodological evolution of peripheral immune cell phenotyping in Parkinson's disease. A. Preferred Reporting Items for Systematic reviews and Meta-Analyses extension for Scoping Reviews (PRISMA-ScR) flow diagram summarizing the identification, screening, eligibility assessment, and inclusion of peripheral immune cell studies in Parkinson's disease (PD). B. Cumulative number of publications over time stratified by study type, distinguishing *in vivo* analyses of immune cells directly isolated from blood and *ex vivo* studies assessing stimulus-evoked activation and resolution responses of PD immune cells in culture. C. Temporal trends in cumulative publications highlighting a progressive shift toward cell-type-resolved analyses of PD peripheral immune populations. D. Cumulative adoption of experimental and analytical techniques used for immune cell profiling in PD, illustrating the transition from early single-analyte immunoassays and targeted biochemical approaches to diversified functional assays and high-dimensional methods. PBMCs, peripheral blood mononuclear cells; APCs, antigen-presenting cells.

Peripheral immune cell profiling in PD first gained momentum in the late twentieth century (Figure 1B, C), primarily through single-analyte antibody-based immunoassays (*e.g.,* ELISA, ELISpot) and targeted biochemical techniques to evaluate gene/protein expression, enzymatic activity, metabolism, and DNA damage (Figure 1D). These early studies focused on broad phenotyping of peripheral blood mononuclear cells (PBMCs), include lymphocytes (T cells, B cells, natural killer (NK) cells and myeloid cells) (Figure 1C). The field expanded steadily through the 2000s, with approximately 50 primary studies published by 2010 that described alterations in the proportions and “*states*” of circulating immune cell subsets isolated directly from blood of PwPD (referred to as *in vivo* studies) (Figure 1B). From 2010 onwards, there was a marked acceleration in the number and scope of studies, reflecting both methodological advances and growing recognition of immune contributions to PD. Research increasingly incorporated cell type-specific analyses of positively or negatively selected subtypes of blood-derived lymphocytes or myeloid cells, including antigen-presenting cells (APCs) and granulocytes as opposed to bulk PBMCs (Figure 1C). During this period, a greater number of functional studies also emerged, employing *ex vivo* cell culture systems to assess immune “*traits*” defined by stimulus-evoked responses (referred to as *ex vivo* studies) (Figure 1B). In parallel, clinical studies using easily accessible methods to quantify major immune populations in the blood (referred to as *rudimentary immunophenotyping*), such as basic flow cytometry (based on cell shape and size), became more common, facilitating translational applications. Rudimentary immunophenotyping has identified reproducible changes in immune cell populations and markers for inflammation in peripheral blood from PwPD (see Table 1 for the list of included studies). Most notable are increases in the neutrophil to lymphocyte ratio (NLR), with ten papers (out of 14 studies that measured NLR) reporting this change relative to healthy donors (Fig. S1).

**Table 1.** List of studies included as *Rudimentary immunophenotyping*.

	Title	References
1	The role of peripheral inflammation-related biomarkers in distinguishing Parkinson's disease	^26^
2	Peripheral blood inflammatory cytokines are associated with rapid eye movement sleep behavior disorder in Parkinson's disease	^27^
3	Differential leukocyte count is associated with clinical phenotype in Parkinson's disease	^28^
4	Peripheral immune profile in drug-naïve dementia with Lewy bodies	^29^
5	Peripheral Immune Profile and Neutrophil-to-Lymphocyte Ratio in Parkinson's Disease	^30^
6	Peripheral Inflammation Is Associated with Dopaminergic Degeneration in Parkinson's Disease	^31^
7	Compared with the monocyte to high-density lipoprotein ratio (MHR) and the neutrophil to lymphocyte ratio (NLR), the neutrophil to high-density lipoprotein ratio (NHR) is more valuable for assessing the inflammatory process in Parkinson's disease	^32^
8	Red cell distribution width levels in Parkinson's disease patients	^33^
9	Investigation of the peripheral inflammation (neutrophil-lymphocyte ratio) in two neurodegenerative diseases of the central nervous system	^34^
10	Inflammation-associated peripheral blood cells and serum lipid levels are associated with Parkinson's disease	^35^
11	Association of inflammatory factors and aging in Parkinson's disease	^36^
12	Lower Lymphocyte Count is Associated With Increased Risk of Parkinson's Disease	^37^
13	Neutrophil-to-lymphocyte ratio and lymphocyte count reflect alterations in central neurodegeneration-associated proteins and clinical severity in Parkinson Disease patients	^38^
14	Peripheral Immune Cell Numbers and C-Reactive Protein in Parkinson's Disease: Results from a Population-Based Study	^39^
15	Comparison of neutrophil-lymphocyte ratio (NLR) in Parkinson's disease subtypes	^40^
16	The increase of carcinoembryonic antigen (CEA), high-sensitivity C-reactive protein, and neutrophil/lymphocyte ratio in Parkinson's disease	^41^
17	Monocyte to high-density lipoprotein cholesterol ratio reflects the peripheral inflammatory state in Parkinsonian disorders	^42^
18	Platelet-to-lymphocyte ratio and neutrophil-tolymphocyte ratio may reflect differences in PD and MSA-P neuroinflammation patterns	^43^
19	Clinical correlation of peripheral CD4+-cell sub-sets, their imbalance and Parkinson's disease	^44^
20	Aging-associated alteration of subtelomeric methylation in Parkinson's disease	^45^
21	A percentage analysis of the telomere length in Parkinson's disease patients	^46^
22	Increase in peripheral CD4 bright + CD8 dull + T cells in Parkinson disease	^47^
23	Calcium influx: An essential process by which *α*-Synuclein regulates morphology of erythrocytes	^48^
24	Prognostic significance of peripheral neutrophils and lymphocytes in early untreated Parkinson's disease: an 8-year follow-up study	^49^
25	Reduction of Peripheral Blood iNKT and *γ*δT Cells in Patients With Parkinson's Disease: An Observational Study	^50^
26	Micellar electrokinetic chromatography with laser induced fluorescence detection shows increase of putrescine in erythrocytes of Parkinson's disease patients	^51^
27	Increased abundance of myeloid-derived suppressor cells and Th17 cells in peripheral blood of newly-diagnosed Parkinson's disease patients	^52^
28	Peripheral Lymphocyte Subsets as a Marker of Parkinson's Disease in a Chinese Population	^53^
29	Increased epigenetic age and granulocyte counts in the blood of Parkinson's disease patients	^54^
30	Clinical characteristics and peripheral T cell subsets in Parkinson's disease patients with constipation	^55^
31	Reduced T helper and B lymphocytes in Parkinson's disease	^56^
32	Electron transfer complexes I and IV of platelets are abnormal in Parkinson's disease but normal in Parkinson-plus syndromes	^57^

Technological diversification was a defining feature of the past 15 years (Figure 1D). Classical immunoassays were complemented by approaches interrogating cellular functions, such as phagocytosis, proliferation, apoptosis and cytotoxicity, as well as unbiased large-scale methodologies, including bulk and single-cell sequencing. Despite this expansion, flow cytometry has remained the primary tool for immune cell profiling in PD, highlighting its central role in the field. Taken together, this longitudinal overview reveals a steady and then rapid growth of research examining peripheral immune cell alterations in PD. The evolution of methodologies has enabled increasingly detailed characterization of immune phenotypes and functions. Synthesizing these developments is essential to identify key findings, methodological disparities, and remaining knowledge gaps.

### Synthesis of demographic and clinical heterogeneity across immune-focused PD studies

Differences in cohort composition, including sample size, sex distribution, age, disease stage/severity, clinical assessments, therapies, and exclusion criteria can influence immune measurements and may partly explain the disparate findings reported in the PD literature. By examining these parameters, we aimed to provide a clearer contextual framework for interpreting immune alterations across studies stratified by study types (*in vivo* versus *ex vivo*) (Figure 2).

**Figure 2.**
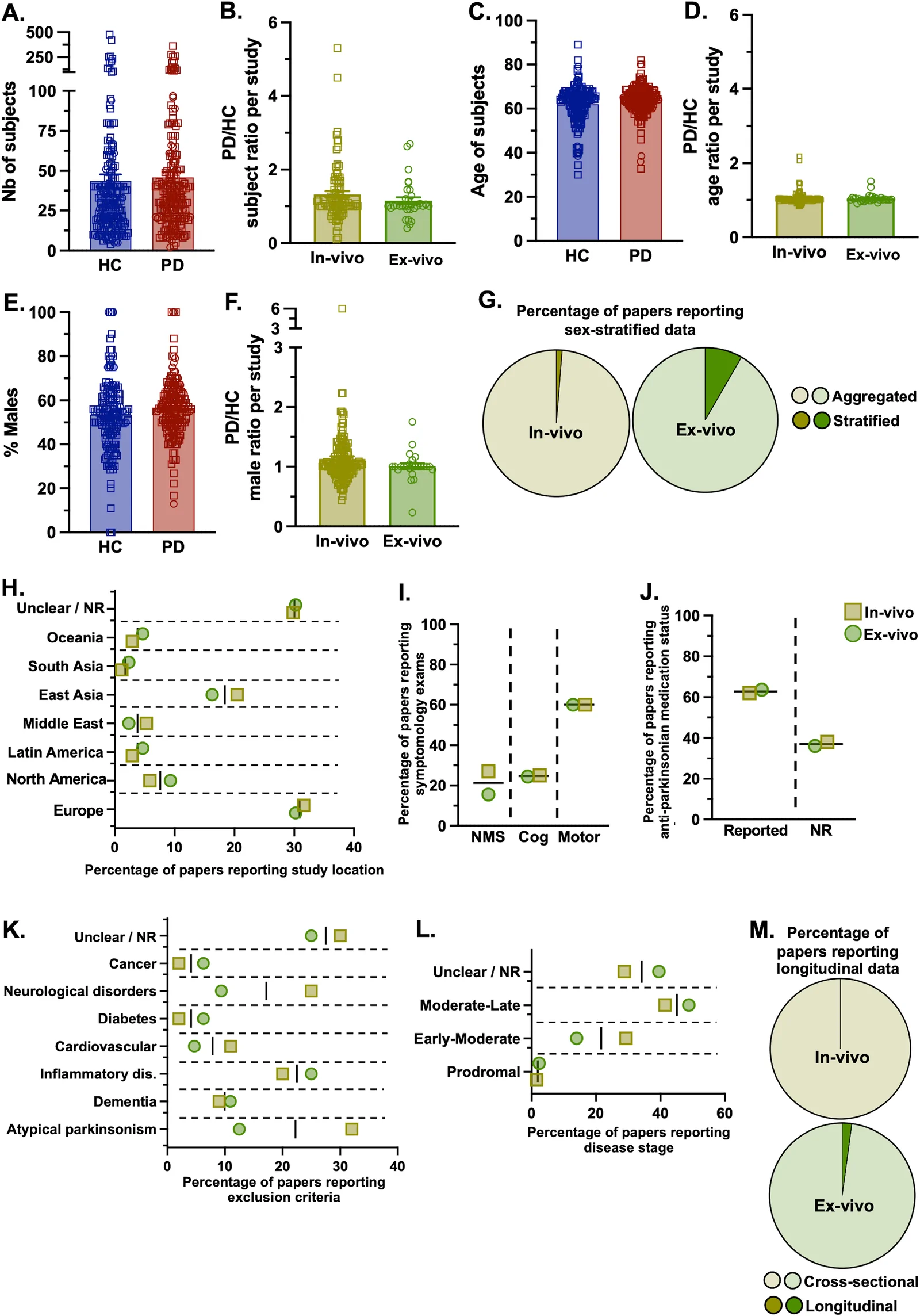
Participant demographics and clinical variables in peripheral immune studies of Parkinson's disease. A–F. Distributions and ratios of cohort size, age, and sex between healthy control (HC) and PD groups across included *in vivo* and *ex vivo* studies. G–M. Frequency of publications reporting sex-stratified analyses, geographic and ethnic origins of study cohorts, clinical symptom assessments, anti-Parkinsonian medication status, exclusion criteria, disease stage and longitudinal data. NR, not reported; NMS, non-motor symptoms; Cog, cognitive.

Analysis of 292 studies (see Tables 2–5 for the list of included studies) revealed substantial variability in both cohort characteristics and the extent of reporting. Overall, study sizes spanned a broad range (<5 to >400 subjects), but the numbers of participants in PD and healthy control (HC) groups were generally comparable across study types (Figure 2A, B). Age and sex distribution were similarly matched between cohorts, with mean ages of 62 and 64 years (Figure 2C, D), and an average male proportion of 53% in HC and 56% in PD groups, respectively (Figure 2E, F). However, sex balance was not always achieved within individual studies, with nearly 46% of *in vivo* studies enrolling proportionally more males in the PD group than in the HC group. Furthermore, despite routinely reporting participant sex, only a small minority of studies performed sex-stratified analyses, with fewer than 2% of in vivo studies and approximately 8% of ex vivo studies reporting immune outcomes separately by sex (Figure 2G). Study cohorts were also geographically diverse (Figure 2H), with ∼30% being of European descent, ∼20% from East Asian countries, and 10% from North America. Fewer than 5% of recruited subjects were either from Latin America, Middle East, South Asia or Oceania. Of note, about a third of studies did not report the ethnicities of their participants.

**Table 2.** List of studies included as *In vivo advanced immunophenotyping* (innate).

	Title	References
1	Inhibition of cathepsin L alleviates the microglia-mediated neuroinflammatory responses through caspase-8 and NF-κB pathways	^58^
2	Reduced Immunosenescence of Peripheral Blood T Cells in Parkinson's Disease with CMV Infection Background	^59^
3	P2X7 receptor/NLRP3 inflammasome complex and *α*-synuclein in peripheral blood mononuclear cells: a prospective study in neo-diagnosed, treatment-naïve Parkinson's disease	^60^
4	Mitochondrial dysfunction and increased glycolysis in prodromal and early Parkinson's blood cells	^61^
5	Transcriptional profile of Parkinson blood mononuclear cells with LRRK2 mutation	^62^
6	Altered NR4A Subfamily Gene Expression Level in Peripheral Blood of Parkinson's and Alzheimer's Disease Patients	^63^
7	Natural killer cells of Parkinson's disease patients are set up for activation: a possible role for innate immunity in the pathogenesis of this disease	^64^
8	Peripheral leukocyte apoptosis in patients with Parkinsonism: correlation with clinical characteristics and neuroimaging findings	^65^
9	Abnormal B-Cell and Tfh-Cell Profiles in Patients With Parkinson Disease: A Cross-sectional Study	^66^
10	Glucocerebrosidase Activity is Reduced in Cryopreserved Parkinson's Disease Patient Monocytes and Inversely Correlates with Motor Severity	^67^
11	Alterations of p11 in brain tissue and peripheral blood leukocytes in Parkinson's disease	^68^
12	Altered dendritic cell subset distribution in patients with Parkinson's disease: Impact of CMV serostatus	^69^
13	Increased Phospho-AKT in Blood Cells from LRRK2 G2019S Mutation Carriers	^70^
14	Characterization of peripheral hematopoietic stem cells and monocytes in Parkinson's disease	^71^
15	Monocyte markers correlate with immune and neuronal brain changes in REM sleep behavior disorder	^15^
16	Systemic activation of NLRP3 inflammasome and plasma *α*-synuclein levels are correlated with motor severity and progression in Parkinson's disease	^72^
17	Measurement of LRRK2 and Ser910/935 phosphorylated LRRK2 in peripheral blood mononuclear cells from idiopathic Parkinson's disease patients	^73^
18	Toll-like receptor expression in the blood and brain of patients and a mouse model of Parkinson's disease	^74^
19	Blood dendritic cell frequency declines in idiopathic Parkinson's disease and is associated with motor symptom severity	^75^
20	Upregulation of APAF1 and CSF1R in Peripheral Blood Mononuclear Cells of Parkinson's Disease	^76^
21	CXCL12 and CXCR4 in the Peripheral Blood of Patients with Parkinson's Disease	^77^
22	Increased Tc17 cell levels and imbalance of naïve/effector immune response in Parkinson's disease patients in a two-year follow-up: a case control study	^78^
23	Regulatory impairment in untreated Parkinson's disease is not restricted to Tregs: other regulatory populations are also involved	^79^
24	Peripheral Blood Immune Cells from Individuals with Parkinson's Disease or Inflammatory Bowel Disease Share Deficits in Iron Storage and Transport that are Modulated by Non-Steroidal Anti-Inflammatory Drugs	^80^
25	Pattern of Mitochondrial Respiration in Peripheral Blood Cells of Patients with Parkinson's Disease	^81^
26	Saposin C, Key Regulator in the Alpha-Synuclein Degradation Mediated by Lysosome	^82^
27	Profiling the Biochemical Signature of GBA-Related Parkinson's Disease in Peripheral Blood Mononuclear Cells	^83^
28	Peripheral innate immune and bacterial signals relate to clinical heterogeneity in Parkinson's disease	^84^
29	Disturbed expression of autophagy genes in blood of Parkinson's disease patients	^85^
30	Expression Profiling of Blood microRNAs 885, 361, and 17 in the Patients with the Parkinson's disease: Integrating Interaction Data to Uncover the Possible Triggering Age-Related Mechanisms	^86^
31	Regulation of myeloid cell phagocytosis by LRRK2 via WAVE2 complex stabilization is altered in Parkinson's disease	^87^
32	RNA-binding disturbances as a continuum from spinocerebellar ataxia type 2 to Parkinson disease	^88^
33	Down-regulation of B cell-related genes in peripheral blood leukocytes of Parkinson's disease patients with and without GBA mutations	^89^
34	Leukocyte glucocerebrosidase and *β*-hexosaminidase activity in sporadic and genetic Parkinson disease	^90^
35	Lysosomal alterations in peripheral blood mononuclear cells of Parkinson's disease patients	^91^
36	Memory consolidation and inducible nitric oxide synthase expression during different sleep stages in Parkinson disease	^92^
37	Involvement of the immune system, endocytosis and EIF2 signaling in both genetically determined and sporadic forms of Parkinson's disease	^93^
38	Elevated alpha-synuclein impairs innate immune cell function and provides a potential peripheral biomarker for Parkinson's disease	^94^
39	Ubiquitin proteasome system in Parkinson's disease: a keeper or a witness?	^95^
40	Altered expression of autophagic genes in the peripheral leukocytes of patients with sporadic Parkinson's disease	^96^
41	MAPK-pathway activity, Lrrk2 G2019S, and Parkinson's disease	^97^
42	Differential expression of splice variant and wild-type parkin in sporadic Parkinson's disease	^98^
43	Beta-adrenoceptor expression on circulating mononuclear cells of idiopathic Parkinson's disease and autonomic failure patients before and after reduction of central sympathetic outflow by clonidine	^99^
44	Understanding LRRK2 kinase activity in preclinical models and human subjects through quantitative analysis of LRRK2 and pT73 Rab10	^99^
45	Autophagic signatures in peripheral blood mononuclear cells from Parkinson's disease patients	^100^
46	Parkin mRNA Expression Levels in Peripheral Blood Mononuclear Cells in Parkin-Related Parkinson's Disease	^101^
47	The regulation of NFKB1 on CD200R1 expression and their potential roles in Parkinson's disease	^102^
48	Supercomplex formation of mitochondrial respiratory chain complexes in leukocytes from patients with neurodegenerative diseases	^103^
49	Immunophenotyping Tracks Motor Progression in Parkinson's Disease Associated with a TH Mutation	^104^
50	A leucine-rich repeat kinase 2 (LRRK2) pathway biomarker characterization study in patients with Parkinson's disease with and without LRRK2 mutations and healthy controls	^105^
51	Orchestration of a blood-derived and ADARB1-centred network in Alzheimer's and Parkinson's disease	^106^
52	A blood-based marker of mitochondrial DNA damage in Parkinson's disease	^107^
53	NF-κB/c-Rel DNA-binding is reduced in substantia nigra and peripheral blood mononuclear cells of Parkinson's disease patients	^108^
54	Alterations in Proteostasis System Components in Peripheral Blood Mononuclear Cells in Parkinson Disease: Focusing on the HSP70 and p62 Levels	^109^
55	Insulin-like growth factor 2 and autophagy gene expression alteration arise as potential biomarkers in Parkinson's disease	^110^
56	Changes in CD163+, CD11b+, and CCR2 + peripheral monocytes relate to Parkinson's disease and cognition	^17^
57	Differential Phospho-Signatures in Blood Cells Identify LRRK2 G2019S Carriers in Parkinson's Disease	^111^
58	Expression of autophagy related genes in peripheral blood cells in Parkinson's disease	^112^
59	Differentially Expressed Circular RNAs in Peripheral Blood Mononuclear Cells of Patients with Parkinson's Disease	^113^
60	An attempt to dissect a peripheral marker based on cell pathology in Parkinson's disease	^114^
61	Subnormal GM1 in PBMCs: Promise for Early Diagnosis of Parkinson's Disease?	^115^
62	Systemic activation of Nrf2 pathway in Parkinson's disease	^116^
63	An Assessment of LRRK2 Serine 935 Phosphorylation in Human Peripheral Blood Mononuclear Cells in Idiopathic Parkinson's Disease and G2019S LRRK2 Cohorts	^117^
64	Elevated In Vitro Kinase Activity in Peripheral Blood Mononuclear Cells of Leucine-Rich Repeat Kinase 2 G2019S Carriers: A Novel Enzyme-Linked Immunosorbent Assay-Based Method	^118^
65	Altered Transcriptional Profile of Mitochondrial DNA-Encoded OXPHOS Subunits, Mitochondria Quality Control Genes, and Intracellular ATP Levels in Blood Samples of Patients with Parkinson's Disease	^119^
66	A compound downregulation of SRRM2 and miR-27a-3p with upregulation of miR-27b-3p in PBMCs of Parkinson's patients is associated with the early stage onset of disease	^120^
67	Autophagy dysfunction in peripheral blood mononuclear cells of Parkinson's disease patients	^121^
68	Expression of the gene coading for PGC-1*α* in peripheral blood leukocytes and related gene variants in patients with Parkinson's disease	^122^
69	Alteration of autophagy-related proteins in peripheral blood mononuclear cells of patients with Parkinson's disease	^123^
70	Interrogating Parkinson's disease LRRK2 kinase pathway activity by assessing Rab10 phosphorylation in human neutrophils	^124^
71	Reduced glucocerebrosidase activity in monocytes from patients with Parkinson's disease	^125^
72	Expression changes of genes associated with apoptosis and survival processes in Parkinson's disease	^126^
73	Dopamine Agonists Exert Nurr1-inducing Effect in Peripheral Blood Mononuclear Cells of Patients with Parkinson's Disease	^127^
74	Peripheral markers in neurodegenerative patients and their first-degree relatives	^128^
75	Alpha-synuclein nitration and autophagy response are induced in peripheral blood cells from patients with Parkinson disease	^129^
76	Expression of clock genes Per1 and Bmal1 in total leukocytes in health and Parkinson's disease	^130^
77	Peripheral blood mononuclear cells from mild cognitive impairment patients show deregulation of Bax and Sod1 mRNAs	^131^
78	Decreased activities of lysosomal acid alpha-D-galactosidase A in the leukocytes of sporadic Parkinson's disease	^132^
79	Oxidative stress in peripheral blood mononuclear cells from patients with Parkinson's disease: negative correlation with levodopa dosage	^133^
80	Oxidative stress level in circulating neutrophils is linked to neurodegenerative diseases	^134^
81	Decreased phagocytic function in patients with Parkinson's disease	^135^
82	Polymorphonuclear leukocyte nitrite content and antioxidant enzymes in Parkinson's disease patients	^136^
83	Neutrophil function, nitric oxide, and blood oxidative stress in Parkinson's disease	^137^
84	Phagocytic cell function in aged subjects	^138^
85	Inflammatory dysregulation of blood monocytes in Parkinson's disease patients	^139^
86	Dysregulation of peripheral monocytes and pro-inflammation of alpha-synuclein in Parkinson's disease	^140^
87	Ex vivo expansion of dysfunctional regulatory T lymphocytes restores suppressive function in Parkinson's disease	^141^
88	Specific immune status in Parkinson's disease at different ages of onset	^142^
89	Dysregulation of mitochondrial and proteolysosomal genes in Parkinson's disease myeloid cells	^143^
90	Sex-based differences in the activation of peripheral blood monocytes in early Parkinson disease	^144^
91	A monocyte gene expression signature in the early clinical course of Parkinson's disease	^145^
92	The Parkinson's disease VPS35[D620N] mutation enhances LRRK2-mediated Rab protein phosphorylation in mouse and human	^146^
93	LRRK2 levels in immune cells are increased in Parkinson's disease	^147^
94	Peripheral-central immune crosstalk in Parkinson's disease and its association with clinical severity	^148^
95	Natural killer cells have an activated profile in early Parkinson's disease	^149^
96	The Impact of Mitochondrial Fusion and Fission Modulation in Sporadic Parkinson's Disease	^150^

**Table 3.** List of studies included as *In vivo advanced immunophenotyping* (adaptive).

	Title	References
1	Alterations of Peripheral Lymphocyte Subsets in Isolated Rapid Eye Movement Sleep Behavior Disorder	^151^
2	The role of immune and inflammatory-related indicators in cognitive dysfunction and disease severity in patients with Parkinson's disease	^152^
3	Increased Levels of Pro-Inflammatory and Anti-Inflammatory Cellular Responses in Parkinson's Disease Patients: Search for a Disease Indicator	^153^
4	Dysregulation of the Adaptive Immune System in Patients With Early-Stage Parkinson Disease	^20^
5	CD4+ regulatory and effector/memory T cell subsets profile motor dysfunction in Parkinson's disease	^154^
6	Reduced Activated T Lymphocytes (CD4+ CD25+) and Plasma Levels of Cytokines in Parkinson's Disease	^155^
7	Inflammation and regulatory T cell genes are differentially expressed in peripheral blood mononuclear cells of Parkinson's disease patients	^156^
8	Peripheral blood lymphocyte phenotypes in Alzheimer and Parkinson's diseases	^157^
9	A correlation study between serum adenosine deaminase activities and peripheral lymphocyte subsets in Parkinson's disease	^157^
10	T-lymphocyte immuno-interferon binding in Parkinsonian patients	^158^
11	T-lymphocyte tumor necrosis factor-alpha receptor binding in patients with Parkinson's disease	^159^
12	T-cell dysregulation is associated with disease severity in Parkinson's Disease	^160^
13	Alterations of T-lymphocyte populations in Parkinson disease	^161^
14	Early-to-mid stage idiopathic Parkinson's disease shows enhanced cytotoxicity and differentiation in CD8T-cells in females	^14^
15	Identification and validation of key molecules associated with humoral immune modulation in Parkinson's disease based on bioinformatics	^162^
16	Changes of T lymphocyte subpopulations and their roles in predicting the risk of Parkinson's disease	^163^
17	T lymphocyte senescence is attenuated in Parkinson's disease	^164^
18	CD4+ T-cell Transcription Factors in Idiopathic REM Sleep Behavior Disorder and Parkinson's Disease	^165^
19	Expression of Transcription Factors in CD4 + T Cells as Potential Biomarkers of Motor Complications in Parkinson's Disease	^166^
20	Abnormalities of age-related T cell senescence in Parkinson's disease	^167^
21	Immortalized Parkinson's disease lymphocytes have enhanced mitochondrial respiratory activity	^168^
22	Effects of peripheral lymphocyte subpopulations and the clinical correlation with Parkinson's disease	^169^
23	Alpha-synuclein induces apoptosis by altered expression in human peripheral lymphocyte in Parkinson's disease	^170^
24	Decrease of the D3 dopamine receptor mRNA expression in lymphocytes from patients with Parkinson's disease	^171^
25	V region T cell receptor repertoire in Parkinson's disease	^172^
26	Beta-adrenergic sensitivity in Parkinson's disease: effect of levodopa treatment	^173^
27	Association between peripheral adaptive immune markers and disease progression in Parkinson's disease	^174^
28	Searching for Biomarkers in the Blood of Patients at Risk of Developing Parkinson's Disease at the Prodromal Stage	^175^
29	Upgraded Methodology for the Development of Early Diagnosis of Parkinson's Disease Based on Searching Blood Markers in Patients and Experimental Models	^176^
30	Peripheral BDNF/TrkB protein expression is decreased in Parkinson's disease but not in Essential tremor	^177^
31	Dopamine Receptor D3 Expression Is Altered in CD4(+) T-Cells From Parkinson's Disease Patients and Its Pharmacologic Inhibition Attenuates the Motor Impairment in a Mouse Model	^178^
32	Neurotrophin Expression in Lymphocytes: a Powerful Indicator of Degeneration in Parkinson's Disease, Amyotrophic Lateral Sclerosis and Ataxia	^179^
33	Lymphocyte vitamin C levels as potential biomarker for progression of Parkinson's disease	^180^
34	Peripheral expression of MAPK pathways in Alzheimer's and Parkinson's diseases	^181^
35	A(2A) adenosine receptors and Parkinson's disease severity	^182^
36	Verification of a Parkinson's disease protein signature in T-lymphocytes by multiple reaction monitoring	^183^
37	Distinctive patterns of DNA methylation associated with Parkinson disease: identification of concordant epigenetic changes in brain and peripheral blood leukocytes	^184^
38	Impact of L-DOPA treatment of patients with Parkinson's disease on mononuclear subsets and phagocytosis in the peripheral blood	^185^
39	Decreased NURR1 and PITX3 gene expression in Chinese patients with Parkinson's disease	^186^
40	Reduced content of *α*-synuclein in peripheral blood leukocytes of patients with LRRK2-associated Parkinson's disease	^187^
41	Verification of expressions of Kir2 as potential peripheral biomarkers in lymphocytes from patients with Parkinson's disease	^188^
42	Peripheral expression of key regulatory kinases in Alzheimer's disease and Parkinson's disease	^189^
43	Increased oxidative damage in peripheral blood correlates with severity of Parkinson's disease	^190^
44	Calcium homeostasis is dysregulated in Parkinsonian patients with L-DOPA-induced dyskinesias	^191^
45	Decreased NURR1 gene expression in patients with Parkinson's disease	^192^
46	Dopamine transporter immunoreactivity in peripheral blood lymphocytes discriminates Parkinson's disease from essential tremor	^193^
47	Peripheral proteasome and caspase activity in Parkinson disease and Alzheimer disease	^194^
48	Increased dopamine content in lymphocytes from high-dose L-Dopa-treated Parkinson's disease patients	^195^
49	Cytogenetic alterations in lymphocytes of Alzheimer's disease and Parkinson's disease patients	^196^
50	Cytogenetic analysis oxidative damage in lymphocytes of Parkinson's disease patients	^197^
51	Reduced dopamine in peripheral blood lymphocytes in Parkinson's disease	^198^
52	Increased expression of dopamine receptors on lymphocytes in Parkinson's disease	^199^
53	Glutamine synthetase activity in patients with Parkinson's disease	^200^
54	Respiratory-chain enzyme activities in isolated mitochondria of lymphocytes from untreated Parkinson's disease patients. Grupo-Centro de Trastornos del Movimiento	^201^
55	Mitochondrial complex I and II activities of lymphocytes and platelets in Parkinson's disease	^202^
56	Peripheral blood cell activities of monoamine oxidase B and superoxide dismutase in Parkinson's disease	^203^
57	Cholinergic muscarinic binding of blood lymphocytes in patients with Parkinson's disease	^204^
58	Evidence for L-dopa incorporation into cell proteins in patients treated with levodopa	^205^
59	Dopamine transporter immunoreactivity in peripheral blood lymphocytes in Parkinson's disease	^206^
60	Involvement of endogenous N-methyl(R)salsolinol in Parkinson's disease: induction of apoptosis and protection by (-)deprenyl	^207^
61	Extraneural cholinergic markers in Alzheimer's and Parkinson's disease	^208^
62	Levodopa therapy reduces DNA damage in peripheral blood cells of patients with Parkinson's disease	^209^
63	Inflammation and IL-4 regulate Parkinson's and Crohn's disease associated kinase LRRK2	^210^
64	Single-cell peripheral immunoprofiling of Alzheimer's and Parkinson's diseases	^211^
65	Multimodal markers of immune activation predict cognitive outcomes in Parkinson's disease	^212^
66	Peripheral Lymphocyte Subsets as a Marker of Parkinson's Disease in a Chinese Population	^53^
67	B lymphocyte responses in Parkinson's disease and their possible significance in disease progression	^213^

**Table 4.** List of studies included as *Sequencing-based immunophenotyping*.

		Title	References
1	B	Accelerated hematopoietic mitotic aging measured by DNA methylation, blood cell lineage, and Parkinson's disease	^214^
2	B	Transcriptome Analysis Reveals a Two-Gene Signature Links to Motor Progression and Alterations of Immune Cells in Parkinson's Disease	^215^
3	B	Discovering Common Pathogenic Mechanisms of COVID-19 and Parkinson Disease: An Integrated Bioinformatics Analysis	^216^
4	B	Multivariate analyses of peripheral blood leukocyte transcripts distinguish Alzheimer's, Parkinson's, control, and those at risk for developing Alzheimer's	^217^
5	B	Stage-dependent phosphoproteome remodeling of Parkinson's disease blood cells	^218^
6	B	SVA Regulation of Transposable Element Clustered Transcription within the Major Histocompatibility Complex Genomic Class II Region of the Parkinson's Progression Markers Initiative	^219^
7	B	Blood transcriptomic signatures associated with molecular changes in the brain and clinical outcomes in Parkinson's disease	^220^
8	B	Coordinated modification in expression levels of HSPA1A/B, DGKH, and NOTCH2 in Parkinson's patients’ blood and substantia nigra as a diagnostic sign: the transcriptomes’ relationship	^221^
9	B	Transcriptome deregulation of peripheral monocytes and whole blood in GBA-related Parkinson's disease	^222^
10	B	Overlap between Central and Peripheral Transcriptomes in Parkinson's Disease but Not Alzheimer's Disease	^223^
11	B	Gene variants and expression changes of SIRT1 and SIRT6 in peripheral blood are associated with Parkinson's disease	^224^
12	B	Transcriptomic profiles in Parkinson's disease	^225^
13	B	Epigenome-wide DNA methylation analysis in siblings and monozygotic twins discordant for sporadic Parkinson's disease revealed different epigenetic patterns in peripheral blood mononuclear cells	^226^
14	B	Parkinson's disease is associated with DNA methylation levels in human blood and saliva	^227^
15	B	Gene Expression Differences in Peripheral Blood of Parkinson's Disease Patients with Distinct Progression Profiles	^228^
16	B	Comparative blood transcriptome analysis in idiopathic and LRRK2 G2019S-associated Parkinson's disease	^229^
17	B	Identification of candidate genes for Parkinson's disease through blood transcriptome analysis in LRRK2-G2019S carriers, idiopathic cases, and controls	^230^
18	B	Involvement of endocytosis and alternative splicing in the formation of the pathological process in the early stages of Parkinson's disease	^231^
19	B	Exon arrays reveal alternative splicing aberrations in Parkinson's disease leukocytes	^232^
20	B	LINE-1 DNA methylation, smoking and risk of Parkinson's disease	^233^
21	B	Aging-related biomarkers for the diagnosis of Parkinson's disease based on bioinformatics analysis and machine learning	^234^
22	B	Analysis of ADORA2A, MTA1, PTGDS, PTGS2, NSF, and HNMT Gene Expression Levels in Peripheral Blood of Patients with Early Stages of Parkinson's Disease	^235^
23	B	Evaluation of Long Non-coding RNA Expression Profiles in Peripheral Blood Mononuclear Cells of Patients with Parkinson's Disease	^236^
24	B	Shared and Unique Disease Pathways in Amyotrophic Lateral Sclerosis and Parkinson's Disease Unveiled in Peripheral Blood Mononuclear Cells	^237^
25	B	METTL14 is decreased and regulates m(6) A modification of *α*-synuclein in Parkinson's disease	^238^
26	B	The expression discrepancy and characteristics of long non-coding RNAs in peripheral blood leukocytes from amyotrophic lateral sclerosis patients	^239^
27	B	Long non-coding RNA ANRIL interacts with microRNA-34a and microRNA-125a, and they all correlate with disease risk and severity of Parkinson's disease	^240^
28	B	Role and Dysregulation of miRNA in Patients with Parkinson's Disease	^241^
29	B	SNCA Hypomethylation in Rapid Eye Movement Sleep Behavior Disorder Is a Potential Biomarker for Parkinson's Disease	^242^
30	B	DNA methylation of the 5′-UTR DAT 1 gene in Parkinson's disease patients	^243^
31	B	DRD2 methylation to differentiate dementia with Lewy bodies from Parkinson's disease	^244^
32	B	Alzheimer's, Parkinson's Disease and Amyotrophic Lateral Sclerosis Gene Expression Patterns Divergence Reveals Different Grade of RNA Metabolism Involvement	^245^
33	B	Pyrosequencing analysis of methylation levels of clock genes in leukocytes from Parkinson's disease patients	^246^
34	B	DNA methylation of imprinted loci of autosomal chromosomes and IGF2 is not affected in Parkinson's disease patients’ peripheral blood mononuclear cells	^247^
35	B	Methylation of *α*-synuclein and leucine-rich repeat kinase 2 in leukocyte DNA of Parkinson's disease patients	^248^
36	B	Long non-coding RNA and alternative splicing modulations in Parkinson's leukocytes identified by RNA sequencing	^249^
37	B	Pyrosequencing analysis of SNCA methylation levels in leukocytes from Parkinson's disease patients	^250^
38	B	Convergence of miRNA expression profiling, *α*-synuclein interacton and GWAS in Parkinson's disease	^251^
39	B	Blood mRNA Expression in Alzheimer's Disease and Dementia With Lewy Bodies	^252^
40	SC	Single-cell RNA sequencing reveals peripheral immunological features in Parkinson's Disease	^253^
41	SC	Global Characterization of Peripheral B Cells in Parkinson's Disease by Single-Cell RNA and BCR Sequencing	^254^
42	SC	A single-cell atlas to map sex-specific gene-expression changes in blood upon neurodegeneration	^255^
43	SC	Mapping the peripheral immune landscape of Parkinson's disease patients with single-cell sequencing	^256^
44	SC	Single-cell transcriptomics reveals the interaction between peripheral CD4+ CTLs and mesencephalic endothelial cells mediated by IFNG in Parkinson's disease	^257^
45	SC	Single-cell transcriptome and TCR profiling reveal activated and expanded T cell populations in Parkinson's disease	^258^
46	SC	Early-to-mid stage idiopathic Parkinson's disease shows enhanced cytotoxicity and differentiation in CD8T-cells in females	^14^
47	SC	The TCR repertoire of *α*-synuclein-specific T cells in Parkinson's disease is surprisingly diverse	^259^
48	SC	Transcriptional analysis of peripheral memory T cells reveals Parkinson's disease-specific gene signatures	^260^

**Table 5.** List of studies included as *Ex vivo immunophenotyping*.

	Title	References
1	Unaltered T cell responses to common antigens in individuals with Parkinson's disease	^261^
2	JKAP relates to disease risk, severity, and Th1 and Th17 differentiation in Parkinson's disease	^262^
3	Human Adipose Tissue-Derived Mesenchymal Stem Cells in Parkinson's Disease: Inhibition of T Helper 17 Cell Differentiation and Regulation of Immune Balance Towards a Regulatory T Cell Phenotype	^263^
4	Parkinson's disease patients have a complex phenotypic and functional Th1 bias: cross-sectional studies of CD4+ Th1/Th2/T17 and Treg in drug-naïve and drug-treated patients	^264^
5	Homologous HSV1 and alpha-synuclein peptides stimulate a T cell response in Parkinson's disease	^265^
6	Dysregulation of peripheral monocytes and pro-inflammation of alpha-synuclein in Parkinson's disease	^140^
7	*α*-Synuclein-containing erythrocytic extracellular vesicles: essential contributors to hyperactivation of monocytes in Parkinson's disease	^266^
8	Toll-like receptor 10 controls TLR2-induced cytokine production in monocytes from patients with Parkinson's disease	^267^
9	Crohn's and Parkinson's Disease-Associated LRRK2 Mutations Alter Type II Interferon Responses in Human CD14(+) Blood Monocytes Ex Vivo	^268^
10	Alterations in Blood Monocyte Functions in Parkinson's Disease	^269^
11	Parkin deficiency modulates NLRP3 inflammasome activation by attenuating an A20-dependent negative feedback loop	^270^
12	Niacin modulates macrophage polarization in Parkinson's disease	^271^
13	Inflammatory dysregulation of blood monocytes in Parkinson's disease patients	^139^
14	Altered regulation of CD200 receptor in monocyte-derived macrophages from individuals with Parkinson's disease	^272^
15	Impaired cytokine production by peripheral blood mononuclear cells and monocytes/macrophages in Parkinson's disease	^273^
16	Immune responses to oligomeric *α*-synuclein in Parkinson's disease peripheral blood mononuclear cells	^274^
17	Probiotics May Have Beneficial Effects in Parkinson's Disease: In vitro Evidence	^275^
18	Herpes simplex virus-1 (HSV-1) infection induces a potent but ineffective IFN-λ production in immune cells of AD and PD patients	^276^
19	Aging, rather than Parkinson's disease, affects the responsiveness of PBMCs to the immunosuppression of bone marrow mesenchymal stem cells	^277^
20	LRRK2-mediated Rab10 phosphorylation in immune cells from Parkinson's disease patients	^278^
21	TLR4 and TLR2 activation is differentially associated with age during Parkinson's disease	^279^
22	Decreased Toll-Like Receptor 2 and Toll-Like Receptor 7/8-Induced Cytokines in Parkinson's Disease Patients	^280^
23	Peripheral cytokines profile in Parkinson's disease	^281^
24	IL-1 beta, IL-2, IL-6 and TNF-alpha production by peripheral blood mononuclear cells from patients with Parkinson's disease	^282^
25	Defective production of interleukin-2 in patients with idiopathic Parkinson's disease	^283^
26	Abnormal immune function of B lymphocyte in peripheral blood of Parkinson's disease	^284^
27	*α*-Synuclein-specific T cell reactivity is associated with preclinical and early Parkinson's disease	^18^
28	Sortilin Expression Levels and Peripheral Immunity: A Potential Biomarker for Segregation between Parkinson's Disease Patients and Healthy Controls	^285^
29	Increased Immune Activation by Pathologic *α*-Synuclein in Parkinson's Disease	^286^
30	Alterations in mitochondrial membrane potential in peripheral blood mononuclear cells in Parkinson's Disease: Potential for a novel biomarker	^287^
31	G1/S Cell Cycle Checkpoint Dysfunction in Lymphoblasts from Sporadic Parkinson's Disease Patients	^288^
32	Targeting cyclin D3/CDK6 activity for treatment of Parkinson's disease	^289^
33	Apoptosis of peripheral blood lymphocytes in Parkinson patients	^290^
34	Oxidative-stress-induced apoptosis in PBLs of two patients with Parkinson disease secondary to alpha-synuclein mutation	^291^
35	*α*-Synuclein orchestrates Th17 responses as antigen and adjuvant in Parkinson's disease	^292^
36	PINK1 is a target of T cell responses in Parkinson's disease	^293^
37	Ambroxol increases glucocerebrosidase (GCase) activity and restores GCase translocation in primary patient-derived macrophages in Gaucher disease and Parkinsonism	^294^
38	Differential Inhibition of LRRK2 in Parkinson's Disease Patient Blood by a G2019S Selective LRRK2 Inhibitor	^295^
39	Reduced expression of the chaperone-mediated autophagy carrier hsc70 protein in lymphomonocytes of patients with Parkinson's disease	^296^
40	Laser modification of the blood in vitro and in vivo in patients with Parkinson's disease	^297^
41	Immune functions in Parkinson's disease lymphocyte subsets, concanavalin A-induced suppressor cell activity and in vitro immunoglobulin production	^298^
42	Effect of LRRK2 protein and activity on stimulated cytokines in human monocytes and macrophages	^299^
43	T cell responses towards PINK1 and *α*-synuclein are elevated in prodromal Parkinson's disease	^16^
44	T cells from patients with Parkinson's disease recognize *α*-synuclein peptides	^300^
45	Peripheral immune cell response to stimulation stratifies Parkinson's disease progression from prodromal to clinical stages	^19^
46	The R1441C-Lrrk2 mutation induces myeloid immune cell exhaustion in an age- and sex-dependent manner in mice	^301^
47	WHOPPA Enables Parallel Assessment of Leucine-Rich Repeat Kinase 2 and Glucocerebrosidase Enzymatic Activity in Parkinson's Disease Monocytes	^302^
48	Parkinson's-Linked LRRK2 and GBA1 Mutations Modulate the Peripheral Immune Response to Pseudomonas aeruginosa	^303^
49	LRRK2 is a negative regulator of Mycobacterium tuberculosis phagosome maturation in macrophages	^304^

Additionally, clinical assessments were inconsistently documented. Although ∼60% of studies reported motor symptom evaluations, far fewer included details of non-motor or cognitive assessments (∼20%) (Figure 2I). Reporting of anti-parkinsonian medication status was also incomplete, with nearly 40% of studies failing to specify treatment details (Figure 2J). Furthermore, exclusion criteria exhibited marked variability (Figure 2K); while atypical parkinsonism, dementia, inflammatory disorders, cardiovascular disease, diabetes, neurological comorbidities, and cancer were noted to different degrees, about a quarter of studies listed no exclusion criteria. These results were largely comparable across *in vivo* and *ex vivo* studies, with a notable exception that neurological disorders and atypical parkinsonism were more frequently excluded in *in vivo* studies. Beyond these factors, disease stage/severity was also unevenly captured across studies. Cohorts ranged from prodromal individuals (diagnosed with REM sleep behaviour disorder, RBD) (<2%) to those with early-to-moderate PD (∼20%) and moderate-to-late PD (∼45%), whereas a sizeable proportion of studies provided unclear or no information on disease stage/severity (∼35%) (Figure 2L). Of note, for our analysis, and in keeping with the literature, we categorized patients with “early to moderate stages” as patients with disease duration <5 years and/or manifesting symptoms/signs observed early in the disease according to established clinical exams, such as H&Y scales I/II, Webster ratings <8, Braak stages I/II or UPDRS-III ratings <18. Within each category, the percentage of participants recruited varied substantially, reflecting inconsistent sampling strategies and highlighting another dimension of clinical heterogeneity that may influence immune readouts. While the vast majority of studies rely on cross-sectional designs, only about 2% of *ex vivo* studies conducted longitudinal immune profiling and none of the *in vivo* studies (Figure 2M).

Overall, these data suggest that the field has achieved consistency in reporting and matching age and sex across cohorts, although remains variable in documenting key demographic and clinical variables, including ethnicity, disease stage/severity, treatment status, exclusion criteria, sex-stratified analyses and longitudinal follow-up. This uneven reporting reveals persistent gaps in standardization in peripheral immune research in PD.

### Overview of in vivo studies illustrating peripheral immune states in PD

To characterize methodological trends in peripheral immune cell profiling in *in vivo* studies, we examined key steps from sample acquisition to data analysis across 163 PD studies. These were stratified according to the primary immune populations analyzed – innate or adaptive (see Tables 2 and 3 for the list of included studies). Notably, although 100 studies assessed both innate and adaptive immune cells, they were categorized based on the immune cell type(s) emphasized in the major conclusions. PBMCs were typically obtained by density-gradient centrifugation, aliquoted, and either analysed immediately (fresh) or cryopreserved (frozen) for future downstream assays (Figure 3A). Although anticoagulants are required to preserve cell integrity and ensure successful isolation of PBMCs, their use can influence immune function. We thus compared the choice of anticoagulants reported in *in vivo* studies. Most reports did not specify the reagent used regardless of immune cell types assessed, while EDTA was the most common among those that did (Figure 3B). The use of fresh *versus* cryopreserved PBMCs was also evaluated. *Adaptive* studies more often relied on freshly processed samples, while *innate* studies showed roughly equal distribution between frozen and fresh material, with ∼10% of all studies lacking sufficient information to determine sample state (Figure 3C). Reporting of centrifugation conditions across both density-gradient separation and subsequent wash steps showed that most papers documented g-force rather than revolutions per minute (RPM) and over half did not fully report spin parameters (Figure 3D). Moreover, despite the potential impact of storage conditions and preparation steps on cell viability and yield, more than half of the studies omitted or incompletely described these features (Figure 3E), which impairs the evaluation of the cell quality, and thus the data obtained, during the analysis. The importance of ensuring the processing pipeline is operator and site independent cannot be underscored enough. Several groups have indeed investigated the extent to which this could be achieved by deep immunophenotyping control PBMCs at two different sites^302^ followed up by *ex vivo* studies on immune inducibility of idiopathic and genetic PD (LRRK2 and GBA1 variants) PBMCs from PD cryorepositories in response to inflammatory stimuli or pathogens.^303,305^

**Figure 3.**
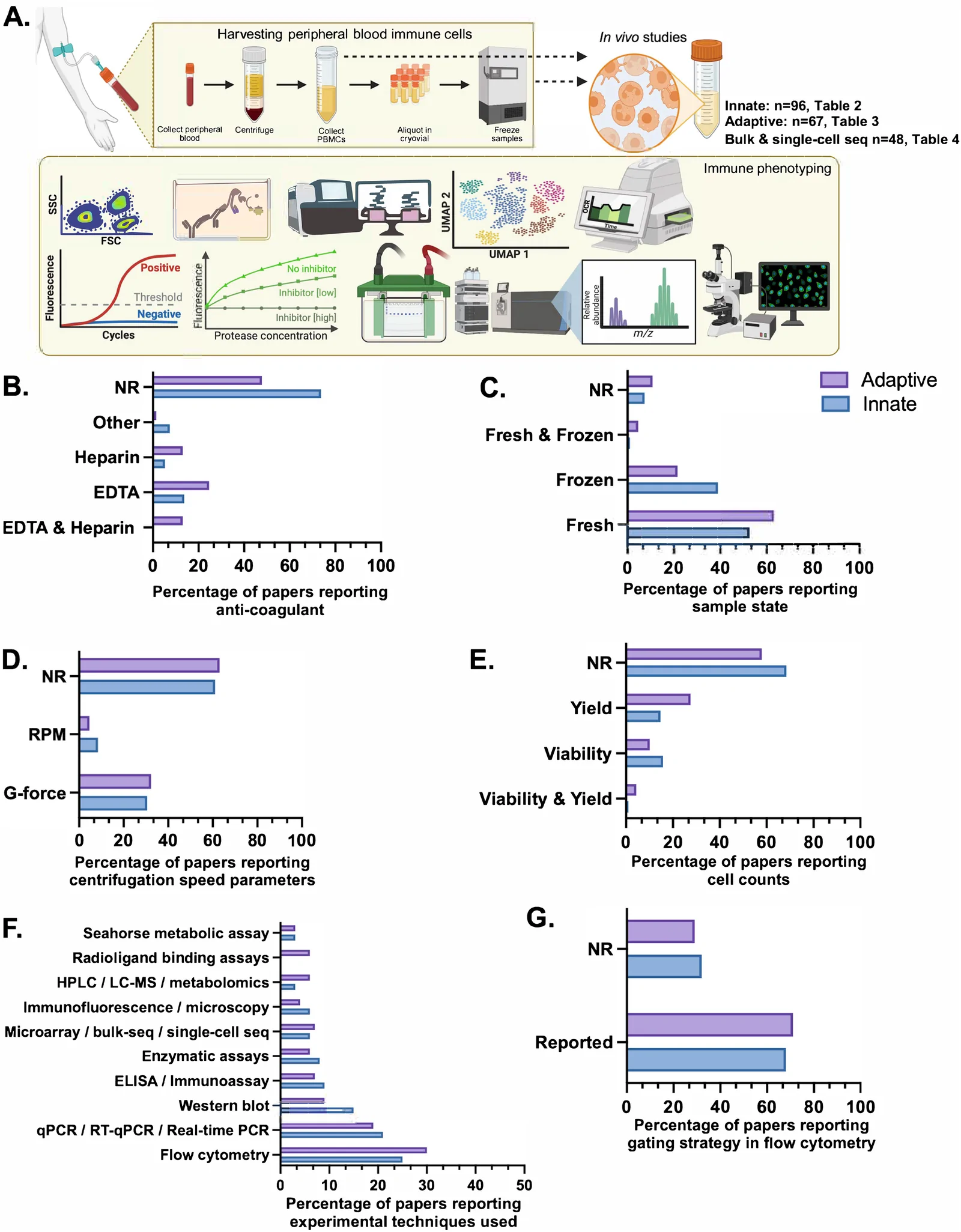
Methodological landscape of *in vivo* peripheral immune cell profiling in Parkinson's disease. A. Schematic representation of experimental workflows in *in vivo* PD peripheral immune research, spanning blood collection, PBMC isolation, and downstream immune phenotyping using molecular, biochemical, cytometric, metabolomic, imaging-based and sequencing approaches. The number and associated list of studies interrogating innate, adaptive, or sequencing-based immune phenotypes are indicated. B–G. Reporting of anticoagulants for blood collection, sample handling practices, centrifugation parameters for PBMC isolation, cell yield and viability measurements, experimental platforms for immune characterization, and gating strategies in flow cytometry-based *in vivo* PD studies, stratified by immune population focus (innate *versus* adaptive). RPM, revolutions per minute (RPM); G, g-force.

We also noted a wide array of analytical platforms that was applied to assess immune *states*. Flow cytometry was the dominant approach, followed by qPCR, Western blotting, and ELISA performed on immune cell lysates and/or supernatants, whereas advanced proteomic, metabolic or imaging assays were less commonly utilized (Figure 3F). Among flow cytometric studies, documentation of gating strategies was inconsistent, with roughly one-third of *in vivo* studies failing to present gating profiles (Figure 3G). Furthermore, studies varied in gating strategies. For instance, in the case of innate cells, some use live/dead discrimination followed by size and granularity parameters for the inclusion gate, whereas others define gates using myeloid-specific cell surface markers such as CD64, TLR2, or CD45 combined with negative selection for markers like CD3, CD19, CD56, and CD66b. Importantly, certain molecules used for classification are also subject to proteolytic cleavage from the surface, as such disparities in protocol or antibodies might result in differences in detected proteins and definition of subpopulations.

In addition to hypothesis-driven methods, 48 studies implemented unbiased, sequencing-based immune profiling, including bulk RNA sequencing (RNA-seq), long non-coding RNA-seq (lncRNA-seq), epigenomic and proteomic analyses, as well as single-cell RNA-seq, with four of these studies also incorporating T-cell and B-cell receptor sequencing (TCR/BCR-seq) (Figure 3F and 4A top panel; see Table 4 for the list of included studies). Among the nine single-cell RNA-seq studies, we extracted the number of cells analyzed and the quality-control parameters applied (Figure 4A, bottom panel) as the rigor and interpretability of sequencing studies depend in part on sampling depth and preprocessing strategies. We observed considerable variability in cell counts, with most studies analyzing between 10,000 and 100,000 cells, and only two exceeding 100,000 cells. In contrast to this heterogeneity in sampling depth, preprocessing practices were comparatively consistent, with seven of nine studies reporting the application of standard quality-control measures including removal of low-quality cells based on mitochondrial transcript reads (typically > 5–20%) and doublet detection using tools such as scDblFinder or DoubletFinder. In droplet-based single-cell RNA-seq experiments, background contamination from ambient RNA is common, arising from both encapsulated cells and extracellular RNA released during cell lysis. Nevertheless, only two studies reported correcting for ambient RNA contamination using SoupX. To summarize cell type-specific aberrant pathways, we extracted major GO and KEGG biological processes reported in nine single-cell RNA-seq studies. Altogether, 118 pathways were described as upregulated and 9 as downregulated in PD compared to matched controls. We then compiled dysregulated pathways denoted in at least two independent studies and mapped to specific immune cell types, including classical monocytes, cytotoxic NK cells, mucosal-associated invariant T cells (MAIT), CD4+ and CD8+ effector T cells (CTLs), CD4+ and CD8+ memory T cells (TEM/TEMRA), and B cells (Figure 4B). Among the shared altered biological processes, innate immune response, T-cell receptor signaling/activation, and leukocyte migration/chemotaxis were repeatedly reported within the same cell types.^14,253,254,256–258,260^ Notably, cytotoxic NK and CD8+ TEM/TEMRA populations were most frequently implicated, with convergent evidence pointing toward heightened activation states in PD.

**Figure 4.**
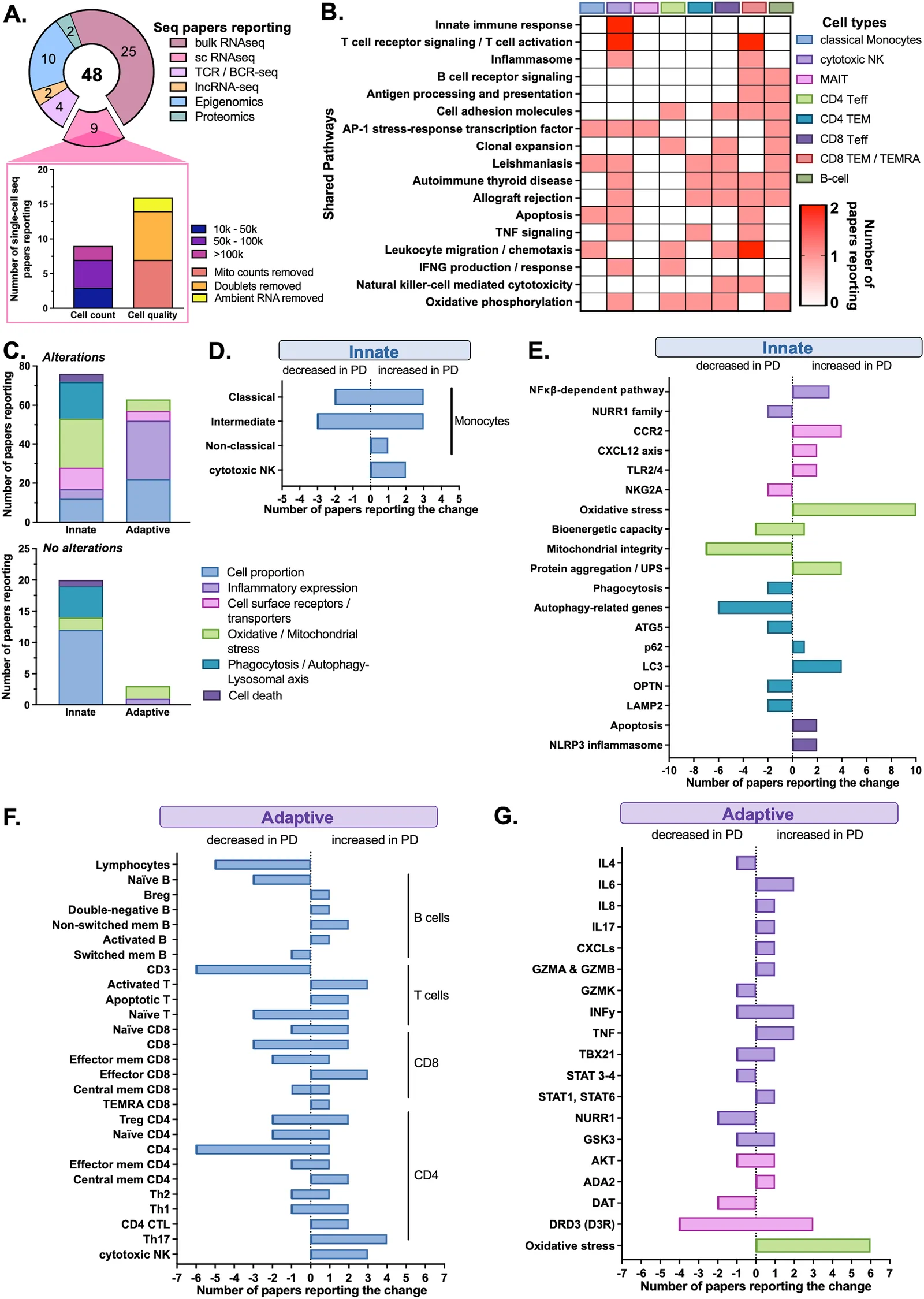
Peripheral immune states uncovered by *in vivo* phenotyping in Parkinson's disease. A. Pie chart depicting the total number of studies employing sequencing-based immune profiling of PBMCs in PD, stratified by omics modality. Shown in the inset bar graph are the methodological features of the nine single-cell RNA sequencing (RNA-seq) studies, including the number of cells analyzed (stratified as 10,000–50,000; 50,000–100,000; and >100,000 cells) and reported quality-control measures (removal of cells with high mitochondrial transcript content, doublet detection, and ambient RNA correction). B. Heatmap summarizing shared GO/KEGG pathways described as dysregulated in specific immune cell populations across published single-cell RNA-seq studies. Only pathways noted in at least two independent studies are presented; color intensity reflects the number of studies reporting each pathway within a given cell type. C. Synthesis of the number of *in vivo* publications describing PD-associated alterations (top) or no alterations (bottom) in terms of cell proportions and distinct molecular features, stratified by innate and adaptive immune populations. D–G. Reporting of directionality and frequency of reported changes in cell proportions and molecular features of either innate or adaptive immune cell subsets in PD. RNA sequencing, RNA-seq; TCR, T-cell receptor; BCR, B-cell receptor; lncRNA-seq, long non-coding RNA-seq; NK, natural killer; MAIT, mucosal-associated invariant T cells; Teff, effector T cells; TEM, effector memory T cells; TEMRA, terminally differentiated effector memory cells re-expressing CD45RA; IFNG, interferon gamma; TNF, tumor necrosis factor; CCR2, chemokine receptor 2; iNOS, inducible nitric oxide synthase; COX2, cyclooxygenase-2; NF-κB, nuclear factor kappa-light-chain-enhancer of activated B cells; NURR1, nuclear receptor-related 1; CXCLs, CXC motif chemokines; TLR2/4, toll-like-receptors 2 & 4; NKG2A, killer cell lectin-like receptor C1 or KLRC1; UPS, ubiquitin-proteasome system; ATG5, autophagy-related protein 5; p62, sequestosome-1 or SQSTM1; LC3, microtubule-associated protein 1 light chain 3; OPTN, optineurin; LAMP2, lysosomal-associated membrane protein 2; NLRP3, NOD-, LRR-, and pyrin domain-containing protein 3; Mem, memory; Treg; regulatory T cells; Th, T helper cells; MDSCs, myeloid-derived suppressor cells; IL, interleukin; TBX21, T-box transcription factor; STAT, signal transducer and activator of transcription; GSK3, glycogen synthase kinase-3; AKT, serine/threonine kinase or PKB; ADA2, adenosine deaminase 2; DAT, dopamine transporter; DRD3 / D3R, dopamine D3 receptor.

Next, we synthesized immune *state*-level reporting across *in vivo* studies to evaluate how often PD-associated changes were described in terms of cell proportions and/or molecular features, encompassing inflammatory gene/protein expression, cell surface receptors/transporters, oxidative/mitochondrial stress, phagocytosis/autophagy-lysosomal axis and cell death, and stratified by study types (Figure 4C, upper graph), including those reporting no detectable differences (Figure 4C, lower graph). Within the innate immune compartment (see Table 2 for the list of included studies), the monocyte frequencies were often unchanged (Figure 4C, lower graph in blue) although few studies reported shifts within monocyte subsets, most commonly either an increase or a reduction of classical (CD14^high^CD16^−^) and intermediate subsets (CD14^low^CD16^−^) and in one case an expansion of non-classical monocytes (CD14^−^CD16^+^) (Figure 4D). Beyond the monocyte population, the changes in the cellular composition of other innate cells are less studied, with one study reporting a reduction in frequencies of both myeloid and plasmacytoid dendritic cells (m- and p-DCs), particularly in newly diagnosed patients.^75^ While another study showed that cytomegalovirus status in PD affects mDC subpopulations,^69^ highlighting infections as environmental modulators of PD immunity. Moreover, the finding that higher proportions of cytotoxic NK cells are described in PD *versus* HC is reproduced in five *in vivo* studies (Figure 4D, F).

These proportional changes were often paralleled by molecular alterations in innate cells that point toward enhanced inflammatory potential and organelle dysfunction, including mitochondria and lysosomes (Figure 4E) with some describing no alterations (Figure 4C, lower graph in green and teal). Across eight studies, monocytes from PwPD exhibit higher expression of cell surface of immune sensors previously associated with neurodegeneration (TLR2/4, TREM2), and receptors involved in immune recruitment and infiltration (CXCR4, CCR2 and CD11b), as well as in antigen presentation (HLA-DR) (Figure 4E; in pink). Three studies also documented increased expression of downstream inflammatory mediators or pathway components, including iNOS, COX-2, TNF and NFκB (Figure 4E; in light purple), reinforcing a shift toward heightened activation states with possible involvement of NLRP3-mediated inflammasomes. Notably, although PD innate immune cells seemed dysregulated and prone to enhanced inflammatory responses, these features did not consistently translate into consistent increased secretion of pro-inflammatory cytokines following stimulation in culture (Figure 5I, J). This might be explained by several mechanisms, such as compromised vesicular compartment, which may be directly related to a disturbance of the autophagy-lysosomal axis, and/or disturbed energy status, comprising nearly 60% of publications reporting dysregulated molecular features (Figure 4C; in teal and green, respectively). PBMCs from PwPD exhibit altered expression of key autophagy-lysosomal genes (ATG5, p62, LC3, OPTN and LAMP2) (Figure 4E; in teal), suggesting an impaired autophagic flux that may hinder chaperone-mediated activities and autophagosome-lysosome fusion leading to the accumulation of autophagic vacuoles. Moreover, lysosomal enzymes have also been investigated in several studies with reports showing either an increase (Cathepsin D/L) or decrease (*α*-D-galactosidase, glucocerebrosidase, *β*-galactosidase, Prosaposin) in circulating immune cells from PwPD depending on the proteins being examined and specific cell type.^58,82,132^ Intriguingly, the amounts of certain lysosomal enzymes are found associated with levels of either *α*-synuclein or inflammatory markers in PD.^58,82,83^ Additionally, twenty-five studies elucidated that PBMCs from PwPD show evidence of mitochondrial impairments, exemplified by elevated ROS production, reduced bioenergetic capacity and disrupted mitochondrial integrity, including mitochondrial content, SOD activity and mitochondrial supercomplex formation (Figure 4E; in green). Conversely, the evidence for increased cell death is rather sparse, with four studies involving both apoptotic and NLRP3-mediated pyroptotic pathways (Figure 4E; in dark purple). Although the impact on cellular viability is less understood, it is important to note that these metabolic alterations could lead to largely different functionalities and efficiencies of innate immune cells.

**Figure 5.**
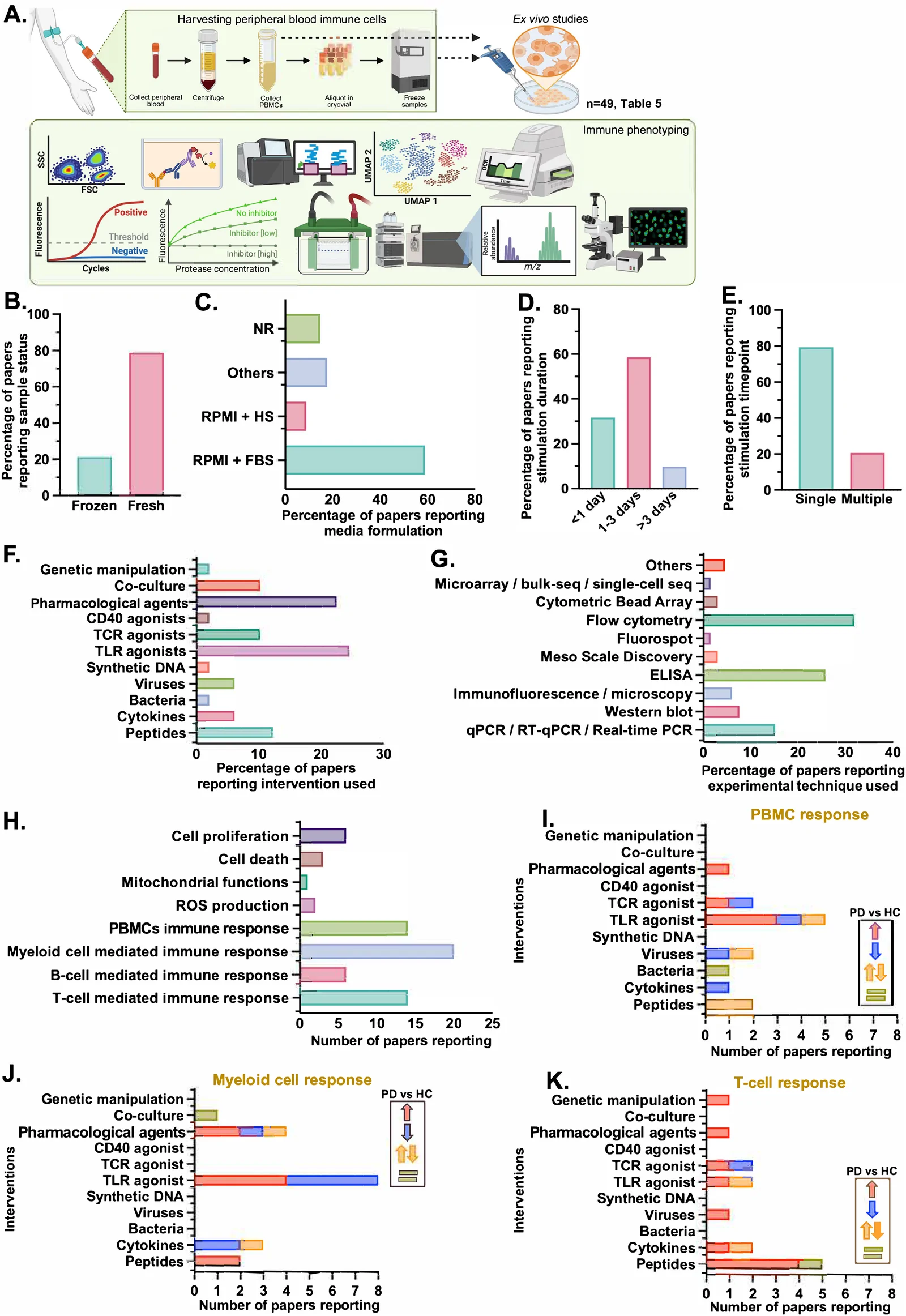
Methodological landscape and immune traits uncovered by *ex vivo* peripheral immune cell profiling in Parkinson's disease. A. Schematic representation of experimental workflows in *ex vivo* PD peripheral immune research, spanning blood collection, PBMCs isolation, cell culture conditions, and downstream immune phenotyping using molecular, biochemical, cytometric, metabolomic and imaging-based approaches. The number and associated list of studies are indicated. B–G. Reporting of sample handling practices, culture media formulations, duration and frequency of stimulation, types of intervention and experimental platforms for immune characterization. H. Illustration of the number of publications documenting the major functional immune changes between HC and PD groups. I–K. Reporting of alterations in PBMC, myeloid cell- and T cell-mediated responses stratified by intervention type. Cell responses indicate the various cytokine outputs, including IFN*γ*, TNF, IL-17A, IL-1*α*, IL-1*β*, IL-2, IL-5, IL-6, IL-8, IL-10. The color indicates the directionality of changes in PD relative to HC. HS, human serum; FBS, fetal bovine serum; TCR, T-cell receptor; ELISA, enzyme-linked immunosorbent assay; PCR, polymerase chain reaction; ROS, reactive oxygen species.

Since LRRK2 is highly expressed in neutrophils, monocytes,^124^ but also B cells,^306^ fifteen *in vivo* studies have utilized blood immune cells to study LRRK2-related changes. Nine of them evaluated LRRK2 kinase activity and explore LRRK2 as a biomarker (Figure S2). The authors reported increased LRRK2 activity in genetic G2019S-LRRK2 PD, while data on LRRK2 activity or expression in sporadic PD was contradictory. Three articles studied LRRK2-related molecular pathways and another three investigated the role of LRRK2 in cellular processes such as phagocytosis and endocytosis although they were not particularly focused on PwPD (Figure S2).

The adaptive immune compartment exhibited similarly heterogeneous yet some reproducible patterns of dysregulation across studies, with findings spanning both cell proportions and molecular features (Figure 4C, see Table 3 for the list of included studies). In terms of cell proportions, overall lymphocyte frequencies were reduced across studies, with T cells (CD3+) reported to be less abundant in PD (Figure 4F). Fewer studies have described B-cell abundance in PD relative to T lymphocytes albeit such studies consistently documented a decrease in naïve B cells concomitant with an increase in specific subsets of regulatory (CD5+ CD19+), double-negative (CD27- IgD-), non-switched memory (CD27+ IgM+ IgD+) and activated (TNF+ CD19+) B cells. By contrast, T-cell populations showed a mixed profile, with changes in cell proportions varying depending on the specific subtypes being assessed, and even across studies. Despite the heterogeneity in T lymphocyte findings, 11 studies described a higher abundance of cytotoxic or pro-inflammatory effector CD8 and/or CD4 subsets in PD, whereas the data examining naïve T-cell populations is more contradictory between studies. These emerging patterns highlight a recurring theme across studies – an expansion of activated lymphocyte populations. Potential skewing toward cytotoxic/pro-inflammatory T-cell programs further aligns with various reports on molecular features indicating elevated expression of IL-6, TNF, IFN-*γ* in PD lymphocytes (Figure 4G; in light purple). However, several transcription factors central to T-cell differentiation (TBX21, STAT1/3/4/6) showed more nuanced results with very few papers interrogating these proteins. Furthermore, dopamine-related markers, including DRD3 and DAT, also differed between PD and matched controls across studies (Figure 4G; in pink), suggesting an impaired dopaminergic signaling in circulating lymphocytes. Increased frequency of DAT+ PBMCs is associated with worsening motor score indicative of disease relevance.^104^ Other aberrant adaptive immune *states* include markers of oxidative stress, which are consistently upregulated in PD and analogous to observations in innate immune studies (Figure 4E, G; in green).

Together, these observations underscore substantial methodological heterogeneity, but also reveal convergent immune signatures, marked by altered subset frequencies and shifts in molecular features that suggest a primed inflammatory activity across both innate and adaptive immune compartments in PD.

### Overview of ex vivo studies illustrating peripheral immune traits in pd

To further contextualize immune findings, we examined methodological characteristics of studies employing *ex vivo* assays to interrogate peripheral immune cell functions in PD (see Table 5 for the list of included studies). These experiments involve isolating PBMCs from patients and healthy donors, followed by culture and stimulation under defined conditions, and then application of a plethora of immune phenotyping approaches (Figure 5A). Sample handling practices varied, with most studies using freshly processed cells rather than cryopreserved samples (Figure 5B). Culture conditions were inconsistently described; approximately 60% used RPMI supplemented with fetal bovine serum (FBS), a smaller proportion employed human serum (HS) or other formulations, and roughly 20% did not specify media composition (Figure 5C). Stimulation protocols also differed, with the majority applying 1–3 days of exposure, about 30% performing acute stimulations of less than 24 h, and only a few extending beyond 3 days (Figure 5D). Most studies analyzed a single time point rather than multiple sampling intervals (Figure 5E), limiting insight into dynamic immune responses. In some studies, specific populations are either positively or negatively selected and then plated *ex vivo* in comparison to plating bulk PBMCs in which the presence of multiple cell types is likely to influence the phenotype of specific subsets of cells being studied.

A wide range of stimuli were employed, with toll-like receptor (TLR) agonists and other target-specific pharmacological agents (such as NFAT, ATP and NLRP3 inhibitors, as well as inflammasome and redox state agonists) being the most common interventions, followed by co-culture systems, T-cell receptor (TCR) agonists and synthetic peptide pools. Fewer than ten functional studies applied genetic manipulations, CD40 agonists, synthetic DNA, viruses, bacteria or cytokines (Figure 5F). Correspondingly, a variety of analytical techniques have been utilized to evaluate functional outcomes. Flow cytometry and ELISA dominated methodological approaches, while multiplex platforms, qPCR, Western blotting, and high-throughput sequencing were less commonly performed (Figure 5G). Functional readouts primarily centered on general cytokine production by PBMCs, followed by assays probing T cell- and myeloid cell-mediated responses (Figure 5H). Other measures included assessments of cell proliferation, cell death, mitochondrial function, reactive oxygen species (ROS) production and B-cell mediated responses. Further stratification of functional readouts by intervention type revealed that both total PBMC and myeloid cell-specific cytokine production were most frequently examined following challenge with TLR agonists (Figure 5I, J), whereas T cell-oriented studies favored stimulation with peptide pools to evaluate antigenic responses (Figure 5K).

Notably, T-cell hyperresponsiveness is the most reproducible immune *trait* in PD (Figure 5K), whereas myeloid and bulk PBMC cytokine outputs show more variable patterns across multiple interventions – ranging from increased, decreased, unchanged or bidirectional changes in PD versus HC (Figure 5J). Stimulation-based assays implicating CD4+ and/or CD8+ T cells revealed exaggerated cytokine production, with repeated reports of elevated secretion of TNF, IFN-*γ*, IL-17A, IL-5 and IL-10 in PD lymphocytes following treatment with mitogens (such as phytohemagglutinin, PHA), *α*-synuclein/viral peptides, Th1-polarizing cytokines, and after JKAP knockdown – a protein tyrosine phosphatase involved in suppressing T-cell immunity. Accordingly, JKAP loss induced cell surface expression of early activation markers CD25 and CD69 in circulating CD4+ T cells from PwPD. While monocytes exhibit both heightened and blunted responses depending on the stimulus, among all cytokines/molecules reported (IL-1*β*, IL-1*α*, IL-8, IL-10, TNF and soluble-CD163), IL-6 production was often measured across all *ex vivo* myeloid cell studies, noting its relevance when assessing innate immunity. Upon comparing interventions, extracellular vesicle stimulation promoted enhanced PD myeloid cell activation in at least two studies, whereas reduced cytokine output occurred in response to mitogens (such as concanavalin A) and TLR2 agonist (Pam3Cys). Notably, both lipopolysaccharide and *α*-synuclein challenges elicited heterogeneous myeloid cell responses across studies, with some reporting increased responses and others decreased responses in PD relative to HC. These bidirectional changes emphasize that the participation of innate immunity in PD is highly context- and ligand-dependent, possibly reflecting differential TLR engagement/expression or altered distribution of classical, intermediate and non-classical subsets which are often not defined in such functional assays. Moreover, bioenergetic changes can also account for the differences as such processes are energetically demanding.

Overall, these findings demonstrate reproducible and robust differences between control and PD PBMCs despite the inherent limitations of methodological differences in cell isolation and handling, *ex vivo* culture conditions, stimulation paradigms, and analytical techniques across studies, which undoubtedly influence and contribute to the reported functional heterogeneity in immune phenotypes in PD.

## Discussion

Across nearly five decades of research, our scoping review underscores the emerging consensus that peripheral immune populations exhibit evidence of dysregulation in PD, with robust differences detectable across multiple sites and numerous assays, although the reported magnitude, direction, and cellular specificity of these alterations vary widely. Our synthesis postulates that this variability is not solely biological but could be amplified by differences in cohort composition, clinical characterization, blood sample handling, anticoagulant selection, cell state, and analytical platforms. Inadequate reporting of critical parameters, including centrifugation conditions, flow cytometry gating strategies, culture media formulations, and viability measures further limits reproducibility and complicates meta-level interpretation. These observations underscore the need for harmonized study protocols and standardized reporting practices across PD immune profiling studies. Of note, despite the implementation of a rigorous and systematic search with carefully constructed keywords, a handful of relevant studies containing additional information exist but were not captured within the databases and search parameters used. To this end, we have manually added such studies curated by experts in the field. This nonetheless highlights a potential gap in the Findable aspect of the FAIR guiding principles,^307^ indicating that despite being present, some research outputs remain difficult to locate.

Reproducible changes in NLR levels identified from studies employing *rudimentary immunophenotyping* are among the notable consistent *in vivo* immune signatures described in this review. However caution is warranted when interpreting NLR data in PD as it is commonly used as a diagnostic marker for inflammation. The sensitivity of immune cells to diverse environmental factors (such as infections) highlights a key challenge when characterizing the role of peripheral immunity in PD. Yet, emerging studies reveal that presenting an immune challenge to PBMCs or enriched populations from peripheral blood uncovers dysfunction in immune responsiveness, which may contribute to the heterogeneity of PD endophenotypes.^19,301,303,304^

Our overview of results from *in vivo* studies unifies key patterns suggestive of an immune system in a “heightened alert” state in PD (Figure 6). These include increased frequencies and activation states of cytotoxic and pro-inflammatory T-cell subsets, altered monocyte subset distributions, and changes in molecular markers of antigen presentation, T-cell engagement, chemotaxis, and innate inflammatory signaling. However, distinguishing disease-related stable immune features from transient *states* that may fluctuate with environmental exposures, stress, circadian rhythms, or medication can be difficult considering the predominance of single-timepoint sampling. This challenge is magnified by the fact that many PD cohorts include individuals on dopaminergic therapies, whereas healthy donors do not. Since dopamine modulates immune signaling,^308^ comparing treated PD patients to untreated controls introduces ambiguity as to whether observed differences, particularly in proteins involved in dopamine transport and/or synthesis, reflect disease mechanisms, treatment effects, or both.

**Figure 6.**
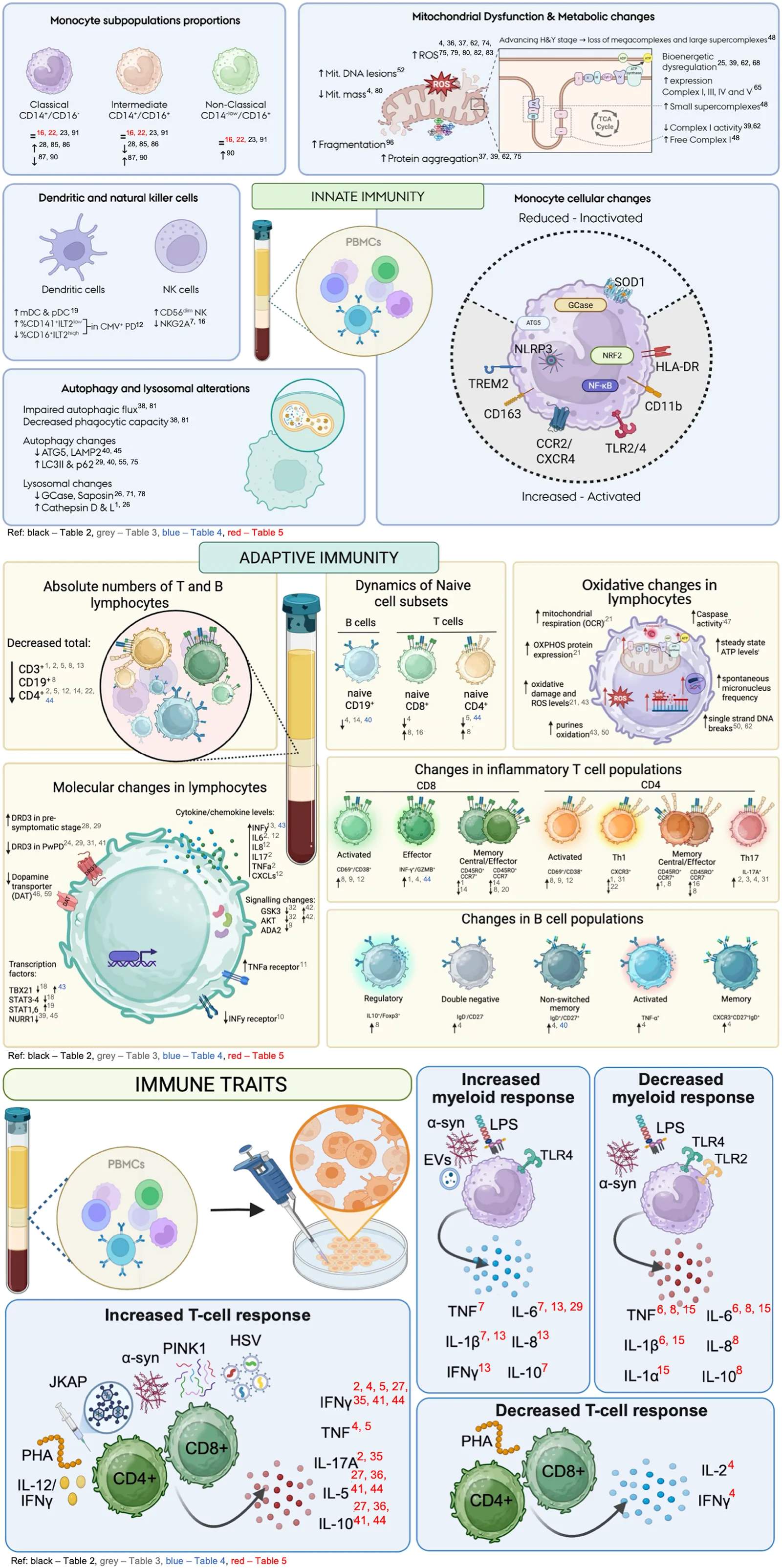
Synthesis of peripheral immune alterations in Parkinson's disease (PD). Graphical summary of key findings from *in vivo* and *ex vivo* studies of peripheral blood immune cells in PD. Innate immune changes include altered monocyte subset distribution, mitochondrial and metabolic dysfunction, impaired autophagy-lysosomal pathways, and dysregulated inflammatory signaling. Adaptive immunity is characterized by reduced circulating lymphocyte numbers, shifts in naïve and memory T- and B-cell subsets, and enhanced pro-inflammatory polarization. Functional assays reveal heterogeneous myeloid and T-cell responses to inflammatory stimuli. Together, these findings support systemic immune dysregulation in PD involving both activation and functional impairment.

To unveil facets of immune perturbations in PD that may be difficult to parse out at baseline, we summarized *ex vivo* studies that offer additional insight into more stable immune phenotypes (Figure 6). A key advantage of these studies is that stimulus-evoked activation and resolution responses, defined as immune *traits*, can be elicited in a controlled experiment regardless of the aforementioned exogenous factors. Indeed, we found that a variety of stimuli similarly induced T-cell hyperresponsiveness in PD, while myeloid cell responses are highly stimulus-dependent, with cytokine release increasing or decreasing in PD depending on the ligand, duration of stimulation, technique utilized, and monocyte subsets present in the culture. Additionally, stimulation-based assays have the potential to increase energetic demand, that may unleash deficits in immune cell bioenergetics, which have been strongly implicated in PD pathogenesis.^309^ This was noted in our review with some *ex vivo* studies reporting metabolic alterations involving cell proliferation, apoptosis, mitochondrial functions and oxidative stress. Consistent with some of these phenotypes, findings from several *in vivo* studies indicate that mitochondria in PBMCs from PwPD may be hyperactive but inefficient, potentially contributing to a toxic cellular environment and chronic inflammation.

Evidence for a hindered autophagy-lysosomal clearance system in PD peripheral immune cells was striking and may point toward compromised intracellular vesicle dynamics. If vesicular trafficking is largely impaired, immune cells could exhibit an inflamed basal transcriptional state while being less capable of secreting cytokines, depending on the assay readout. This model reconciles apparent discrepancies across studies: intracellular cytokine staining using flow cytometry-based methods may show elevated levels, whereas secretion-based assays (such as ELISA) may show blunted cytokine release. Differences in experimental approach could therefore partly underlie the variability in reported functional outcomes. Moreover, alterations in immune activity, vesicular machinery, degradation systems and metabolism are also mirrored through transcriptomics analyses. However, such studies, particularly single-cell omics approaches, remain relatively scarce and underpowered, emphasizing the need for larger, harmonized datasets.

Intriguingly, certain molecular features, such as defective autolysosomes, are shared between idiopathic and genetic forms of the disease as exemplified by GBA mutant carriers showing reduced expression in PBMCs of key lysosomal proteins implicated in autophagy (Saposin C and LAMP2).^83,91^ Similarly, bulk RNA-seq studies detected dysregulated pathways involving mitochondrial function and vesicle trafficking in both idiopathic PD and LRRK2 mutation carriers.^93,310^ Nevertheless, distinct immune signatures involving glucocorticoid receptor and lymphocyte signaling pathways were also identified between these two PD subtypes.^93^ LRRK2 kinase activity in peripheral immune cells provides additional granularity. LRRK2 G2019S carriers have heightened PBMC kinase activity but not in idiopathic PD, while sex-associated differences in LRRK2 activity suggest potential gene-sex interactions shaping immune phenotypes.^118^ A more comprehensive synthesis of subtype-specific immune signatures in PD is thus warranted to understand potential shared and distinct pathogenic mechanisms across genetic backgrounds.

Another major future direction will require systematically resolving how immune changes differ across disease severity. In this review, we listed the proportions of studies enrolling participants at distinct PD stages and noted that most recruited moderate-to-late stage patients, defined by disease duration >5 years and/or clinical measures attributed to low motor performance (H&Y stages III-V, Webster ratings >8, Braak stages III-V, UPDRS-III ratings >18), whereas only a handful have profiled immune changes during the prodromal stage. Several immune features described here vary with disease progression, such as monocyte frequencies and myeloid cell surface markers (*e.g.,* CD11b, CCR2, TLR2/4).^17,84,141^ Many of these abnormalities appear strongest in early, untreated PD and diminish with dopaminergic therapy,^60,75^ raising questions about whether treatment and/or disease severity modulates immune signaling or whether chronic disease leads to immune exhaustion. Meanwhile, other receptors are reported to increase with time as the disease progresses, such as HLA-DR and soluble CD163, although it is unclear if their signaling efficiency remains at later stages.^17,84,141^ Longitudinal studies in treatment-naïve cohorts or prodromal stage are essential to disentangle these possibilities.

Taken together, the literature paints a picture of a peripheral immune system that is activated, imbalanced and metabolically stressed in PD, particularly early in the disease course. However, these changes remain obscured by methodological artifacts and by challenges that impede biologically meaningful interpretation. To build reliable immune endophenotypes in PD and guide the development of immune-modulating therapies, a shift is needed toward studies that (i) adopt standardized reporting, (ii) implement unified sample processing workflows, (iii) integrate multimodal immune profiling across diverse and well-characterized cohorts, (iv) interrogate cell type-specific features rather than bulk measures, and (v) distinguish stable immune *traits* from transient immune *states*. These priorities are well aligned with the recent IMMUPARKNET consensus recommendations for clinical immune and inflammatory profiling in PD,^311^ which provide a practical framework for harmonizing study protocols and improving reproducibility across the field. Future integrative reviews comparing immune signatures across genetic subtypes and disease stages, together with longitudinal immune profiling, will further resolve disease heterogeneity and accelerate biomarker discovery and therapeutic development.

## Illustrations

Graphical representations of *in vivo* and *ex vivo* studies were created with BioRender.

## Supplemental Material

sj-tiff-1-pkn-10.1177_1877718X261479505 - Supplemental material for Profiling peripheral immune cells in Parkinson's disease: A scoping review Supplemental material, sj-tiff-1-pkn-10.1177_1877718X261479505 for Profiling peripheral immune cells in Parkinson's disease: A scoping review by Sherilyn Junelle Recinto, Janna E Jernigan Posey, Nadejda Lefter, Zagorka Vitic, Morten Øgelund Overgaard, Lin Liu, Andrew JM Howden, Klaus Eyer, Marina Romero-Ramos, Malú Gámez Tansey and Jo Anne Stratton in Journal of Parkinson's Disease

sj-tiff-2-pkn-10.1177_1877718X261479505 - Supplemental material for Profiling peripheral immune cells in Parkinson's disease: A scoping review Supplemental material, sj-tiff-2-pkn-10.1177_1877718X261479505 for Profiling peripheral immune cells in Parkinson's disease: A scoping review by Sherilyn Junelle Recinto, Janna E Jernigan Posey, Nadejda Lefter, Zagorka Vitic, Morten Øgelund Overgaard, Lin Liu, Andrew JM Howden, Klaus Eyer, Marina Romero-Ramos, Malú Gámez Tansey and Jo Anne Stratton in Journal of Parkinson's Disease

## Data Availability

Raw tabular data is available at DOI:10.5281/zenodo.21636392.
